# The relationship between structural and functional differences of the lumbar multifidus and structural brain morphology in females with non-specific low back pain: An observational study

**DOI:** 10.1371/journal.pone.0337122

**Published:** 2025-12-02

**Authors:** Rahmat Adnan, Evy Dhondt, Lieven Danneels, Tine Willems, Sophie Van Oosterwijck, Jessica Van Oosterwijck

**Affiliations:** 1 Spine, Head and Pain Research Unit Ghent, Department of Rehabilitation Sciences, Faculty of Medicine and Health Sciences, Ghent University, Ghent, Belgium; 2 Faculty of Sports Science and Recreation, Universiti Teknologi MARA, Shah Alam, Malaysia; 3 Pain in Motion International Research Group, www.paininmotion.be,; 4 Research Foundation – Flanders (FWO), Brussel, Belgium; 5 Department of Rehabilitation Sciences and Physiotherapy, Faculty of Medicine and Health Sciences, University of Antwerp, Antwerp, Belgium; Aichi Prefectural Mikawa Aoitori Medical and Rehabilitation Center for Developmental Disabilities, JAPAN

## Abstract

No earlier study investigates the relationship between the structure and function of lumbar multifidus (LM) and gray matter thickness (GMT) and grey matter volume (GMV) in the regional cortical areas that are related to sensorimotor control in patients with NSLBP and in healthy controls (HCs). This study asseses LM morphology such as muscle and fat cross sectional area (CSA), and muscle fat index (MFI) with T1-weighted Dixon. Muscle functional MRI (mfMRI) was used to assess T2-rest and T2-shift of the LM muscle and cortical GMV and GMT derived from high resolution T1-weighted images. Linear mixed models were used to analyze these relationships. This study revealed positive associations between MFI of the LM and GMV of the paracentral and postcentral cortices were found, irrespective of LBP. Positive associations were found between T2-rest of the LM muscle at multiple lumbar levels, GMT (Rostralmiddlefrontal, caudalmiddlefrontal, precentral, superiorpariental, postcentral) and GMV (rostramiddlefrontal, caudalmiddlefrontal, postcentral, supramarginal, rostralanteriorcingulate). GMT of the caudalmiddlefrontal cortex and posteriorcingulate cortex was positively associated with T2-rest at LL L4 of LM and UL L4 of LM, respectively. A significant negative association was observed between the T2-shift of the LM at UL L4 and GMT of the supramarginal cortex. Negative associations were observed between T2-shift of LM at LL L4 and GMV of the rostralmiddlefrontal and paracentral cortex irrespective of LBP. Our result emphasize that altered LM function and structure in NSBLP and HC, and its associations with altered brain structural properties.

## Background

Low back pain (LBP) is a highly prevalent complaint with a high recurrence rate and a high risk of chronification and disability, resulting in costly healthcare and low productivity worldwide [1–3]. At least 85% of the adult population will suffer from LBP at some point in their lives [1,4]. The majority of LBP complaints have been classified as non-specific LBP (NSLBP), which means that the pain cannot be ascribed or attributed to a specific pathology [5]. Up to 10% of acute LBP complaints will develop into chronic NSLBP [6].

Structural neuroimaging in chronic pain patients has suggested that decreases in gray matter areas are related to changes in nociception, sensorimotor function, cognitive and emotion of pain processing [7–10]. Several authors support the view that atrophy of the brain region is attributed to the damage or loss of gray matter [11–14]. Studies show increases as well as decreases in gray matter thickness (GMT) and gray matter volume (GMV) in NSLBP patients [12,15,16]. Such alterations have been observed in the regional rostral-middle-frontal, caudal-middle-frontal, paracentral, precentral, postcentral, superior parietal, and supra-marginal regions, the insula, and the posterior cingulate and rostral-anterior cingulate cortices [17,18]. Alterations in these sensorimotor related areas may be indicative for impaired sensorimotor performance [19,20].

Alterations and dysfunctions in sensorimotor control of the back muscles are observed in patients suffering from NSLBP [21,22]. The LM muscle is important for providing segmental control and function as dynamic stabilizers of the lumbar spine. Alterations in the timing and the amount of activity of the LM in recurrent and chronic NSLBP have been shown using electromyography and muscle functional magnetic resonance imaging (mfMRI) [23–26]. This ongoing impaired muscle activity produces a continuous load on the spinal structures, thereby making the spine susceptible to strain and further injuries [22]. Structural changes of back muscles have also been observed in NSLBP and include fat infiltrations, atrophy, and altered fiber typing (alterations in the metabolic muscle profile) [27–29]. Atrophy of the LM muscle, as well as fat infiltration in this muscle have been observed in chronic NSLBP patients [24]. There is even more compelling evidence that reveals the poor quality of LM muscles at a microscopic level in the absence of atrophy and fat infiltration in patients with recurrent NSLBP [27]. It is clear that muscle structure associated to the muscle function, and pain impacts both [22]. However, we are starting to understand that pain might not only influence local structures but also has the capability to induce changes in the central nervous system. These central changes might be related to the changes observed in the LM as it has already been shown that the activation of the LM is modulated by the excitability of the brain and spinal cord [30], and a modified organization of the motor cortex representation of the LM can occur in NSLBP [31].

The current study aimed to examine whether a relationship exists between the structure and function of the LM muscle and the supraspinal structural gray matter thickness and volume in the brain areas related to sensorimotor control, while the effect of NSLBP compared to healthy controls (HC) was taken into account. It is assumed that the LM muscle structure and function are associated with the thickness and volume of the gray matter cortices in both HC and NSLBP patients. Based on the previous observations of the relation between decreases in gray matter areas and changes in sensorimotor function. Therefore, it is hypothesized that the GMT and GMV of the sensorimotor cortex are 1) positively associated with muscle CSA; 2) negatively associated with the amount of fat in the LM; 3) positively associated with the T2-rest and with the T2-shift of LM muscle, this in HC and in NSLBP. While in NSLBP lower muscle CSA, higher amount of fat infiltrations, lower T2 rest and T2-shift were expected to be associate with lower GMT and GMV due to alterations and disfunctions which occur in this condition, the opposite was expected for HC who have a healthy brain and LM.

## Materials and methods

### Subjects

A total of 29 females (15 NSLBP patients and 14 HC) aged between 18 and 45 participated in this study. Participants were recruited via flyers and posters distributed at the campuses of Ghent University and the University Hospital Ghent, and at private practices of medical doctors and physiotherapists in Flanders (Belgium), as well as social media posts and adverts in newsletters. Participants had to be female since research has demonstrated a significant alterations of lumbar muscle characteristics [32] and brain morphology [33] in women than men; not pregnant or ≥1 year postnatal; speak the Dutch language; not highly active (i.e., professional athletes, intensive athletic training); and have no contraindications for MRI (such as being claustrophobic, having metal or device implants, or being pregnant). Participants who suffered from major depression; obesity (body mass index (BMI) >30); respiratory, metabolic, neurologic, cardiovascular, or inflammatory diseases; or orthopedic disorders which limited movement were not eligible for study participation. Participants with a history of spinal surgery, spinal trauma, severe spinal deformity, or a severe disorder (such as cancer, a cardiovascular event, etc.) were also excluded.

The NSLBP group comprised out of 15 patients suffering from NSLBP. All included patients had to suffer from NSLBP which was defined as pain in the lumbar region that is not attributable to a recognizable, known specific pathology (e.g., infection, tumor, osteoporosis, fracture, structural deformity, inflammatory disorders (e.g., ankylosing spondylitis), radicular pain/syndrome(pain radiating below the knee or clinical signs of nerve root compression) or cauda equina syndrome) [34]. Individuals with a history of spinal surgery, spinal deformities were not eligible for participation in this study. The first onset of the NSLBP complaints had to be of ≥3 months ago.

The HC group comprised of 14 healthy individuals who had experience no pain for at least one year prior to the experiments took place, and had never experienced LBP complaints for more than 24 hours of sufficient severity to consult a physical therapist (PT) or medical doctor (MD).

### Study design and procedure

This observational study took place at the Ghent University between January 2015 till December of 2016 and was approved by the hospital’s ethical committee (EC UZG 2014/0471). All participants witten and verbally informed about the protocol of the study and provided to sign informed consent prior to the commencement of the study. The study comprised of two sessions, namely a first pre-screening session and a second experimental session during which experimental data was collected.

The pre-screening session took place 4–10 days before the experimental session. During this session demographic and pain related data was collected, and fulfillment of the inclusion criteria was examined using self-made questionnaires. A clinical examination was performed during which body height and weight were measured to determine the BMI, and a radicular examination in case there were any indications for neurological involvement to the NSLBP. Furthermore, an individual’s one-repetition maximum (1-RM) for trunk extension from prone position was determined (as described in Fig 4). Patients were informed regarding the procedures taking place during the experimental session and given instructions to refrain from nicotine, alcohol, caffeine, medication with analgesic effects, and vigorous activities prior to that session.

**Fig 1:**
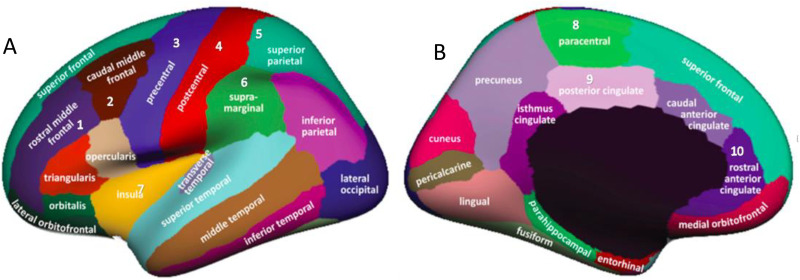
Grey matter regions of interest related to sensorimotor control. Legend: Illustrates the brain regions of interest (ROI) with 1A depicting the cortical ROI: Frontal cortical regions: [1] rostral middle frontal (dorsal lateral prefrontal cortex); [2] caudal middle frontal (premotor cortex); Middle cortical regions: [3] precentral (motor cortex); [4] postcentral (primary somatosensory), [7] insula (primary somatosensory); Posterior cortical regions: [5] superior parietal (secondary somatosensory), [6] supramarginal (secondary somatosensory); 1B showing the subcortical ROI: [8] paracentral (supplementary motor area); [9] posterior cingulate (motor area), [10] rostral anterior cingulate (motor area).

**Fig 2:**
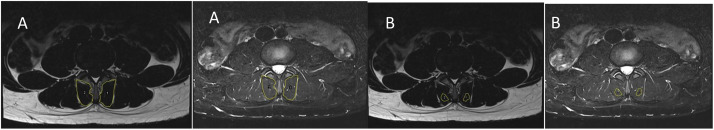
DIXON images of the lumbar multifidus. Legend: Illustrates the DIXON image demonstrating the region of interest (ROI) for the lumbar multifidus muscle, the left imaging showing the fat image and right side showing the water images, with 2A depicting the ROI of the total muscle CSA and B showing the ROI of the lean muscle.

**Fig 3:**
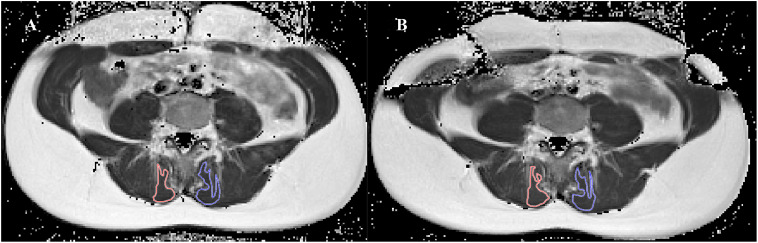
Muscle functional MRI images of the lumbar multifidus. Legend: Illustrates the T2-weighted axial images at lower level-endplate of lumbar 4 (LL L4) demonstrating the region of interest for the lumbar multifidus muscle, with 3A depicting a T2-rest image and 3B showing the T2- after exercise image.

**Fig 4:**
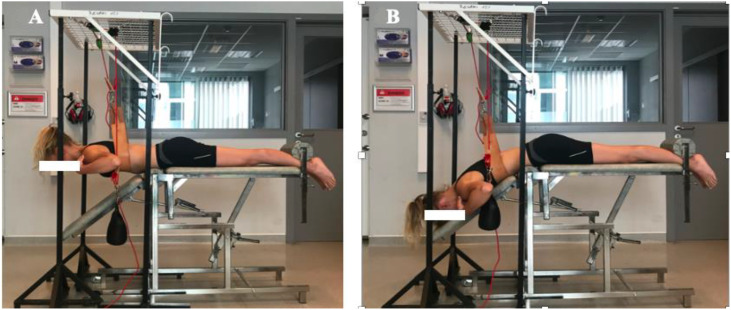
Dynamic-static trunk extension exercise at 40% of 1-RM. Legend: Illustrates the dynamic-static extension exercise with subject raising the trunk to horizontal position (in 2s) and maintaining the horizontal position for 5s (4A), and return (in 2s) to the starting position (4B) of 45° flexion.

During the experimental session a series of self-reported questionnaires related to pain intensity, psychological, health and disability state were filled out. Subsequently, brain structural and muscle structural and functional parameters were evaluated by performing an MRI scan. To acquire images of the brain and muscles, a 3-Tesla Siemens Magneton TrioTim system (Siemens Medical Solutions, Erlangen, Germany) was used. All cost related MRI of this study was supported by the Research Foundation – Flanders (FWO) [12L5619N and 12L5616N] without potential conflict of interest in the text of this manuscript.

### Demographic and disorder related characteristics

Self-developed questionnaires were used to collect information on demographics (age, ethnicity, etc.), NSLBP intensity, and duration. The history (mean and worst globally) and current (during the last 24 hours) NSLBP intensity were recorded using a numeric rating scale (NRS) [35]. Patients also recorded the duration of their symptoms from the first onset of LBP (in months).

The Hospital Anxiety and Depression Scale (HADS) [36,37], and the total score of the Pain Catastrophizing Scale (PCS) [38,39] were used to assess psychological characteristics related to anxiety, depression, and pain catastrophizing.

To examined health status and LBP related disability, the Medical Outcomes Study 36-item short form health survey (SF-36) and the Roland Morris Disability Questionnaire (RMDQ) were used.

### Brain MRI

#### MRI data acquisition.

A 32-channel matrix head coil was used to capture all the brain images. High-resolution T1-weighted images of the brain were acquired using three-dimensional magnetization prepared rapid acquisition gradient echo (MP-RAGE) (repetition time [TR] = 2250 ms, echo time [TE] = 4.18 ms, voxel size = 1 x 1 x 1 mm^3^, FoV = 256 mm, flip angle = 9°, 176 slices, one mm slice thickness, and acquisition time = 5’14”). All T1-weighted anatomical scans were visually checked for overall quality and motion artifacts.

#### FreeSurfer processing.

The high-resolution T1-weighted anatomical scans were analyzed using the FreeSurfer (version 5.3.0) software. These images were analyzed using additional computing resources from the high-performance computing TIER1 cluster available at Ghent University (http://ugent.be/hpc/). The automated approach as described by Fischl et al. [40] was used to extract cortical and subcortical GMT, and GMV, an approach that has produced reliable and accurate results [41]. In brief, these images were processed according to the following steps: [1] removal of non-brain tissue using a hybrid watershed/surface deformation procedure (skull stripping) [42]; [2] automated transformation to Talairach space [3]; segmentation of the subcortical white matter and deep GMV structures [41,43]; [4] intensity normalization [44]; [5] tessellation of the boundary between GM and white matter, and automated topology correction [45,46]; and [6] surface deformation along intensity gradients for optimal placement of the borders between GM, white matter, and cerebrospinal fluid [47–49]. The cerebral cortex was parcellated (automated parcellation) using the Desikan atlas [50]. While the subcortical cortex was segmentated (automated segmentation) using FreeSurfer [41,42]. Estimates of the total intracranial volume of the left and right hemisphere were also obtained for each subject.

#### Region of interest.

The cortex thickness and volume was extracted from 10 regions of interest (ROIs) reported to be involved in the sensorimotor network based on the Desikan atlas. These cortical and subcortical regions was defined based on observation from the previous studies in NSLBP regarding GMT and GMV alterations [17,18],and their relationships to the sensorimotor control aspects. Specifically, 7 ROIs from the cortical cortex and 3 ROIs from the subcortical cortex were drawn. The following 7 ROIs of the cortical cortex were selected:1] rostral middle frontal cortex [51] and 2] caudal middle frontal cortex [12], 3] precentral cortex [52], 4] postcentral cortex [52], 5] superior parietal cortex [53], 6] supramarginal cortex [54] and 7] insula cortex [55]. The 3 ROIs from the subcortical region include the paracentral cortex [56], posterior cingulate cortex [57] and rostral anterior cingulate cortex [17] (see Fig 1). For each ROI, the GMT and GMV for the right and left hemisphere were separately calculated.

### Muscle MRI

#### Lumbar morphology measurement.

After the brain scan, a T1-weighted two-point DIXON scan was used to evaluate the muscle CSA, fat CSA and MFI of the LM muscle. Participants were placed supine on the MRI table with a foam wedge supporting the legs. A flexible 6-element body coil was positioned in the center of the ventral region at the height of the 4^th^ lumbar vertebra (L4), thus allowing the coil to capture the entire lumbar region. A dorsal standard phased-array spine coil acted as the receiver coil [27].

On the sagittal planes, a slap group of 36 slices, (3 mm slice thickness and 22% oversampling) was positioned at the upper level (UL) of the endplate of L4. The parameter measurements for the two-point Dixon fat/water separation were 320 mm FOV read, 6.59ms TE1, 3.67ms TE2 with 5m 01s acquisition time and a 0.7x0.7matrix. The DIXON scan produced both the fat and water images which were used to calculate the total CSA, muscle CSA, and fat CSA of the LM based on the signal intensities obtained in the Siemens environment. The converted DIXON data were processed by the same researcher who was blinded for the participants LBP status.

The ROI of each separate muscle was drawn on the fat images (Fig 2A). The LM muscle was bilaterally outlined on 2 slices at the height of L4. The total CSA of LM was calculated as the number of voxels in the respective ROI and multiplied by voxel size. The signal intensity for fat (SIfat) and the signal intensity for water (SIwater) for the total LM were also obtained on these ROIs [27]. To further calculate the CSA of the lean muscle tissue, the following formula was applied: “total CSA*(1- total fat index)” [58]. To obtain the CSA of the fat tissue in LM muscle (fat CSA), the lean muscle CSA was subtracted from the total CSA.

To estimate the amount of fat in the lean muscle tissue, the fat fraction in the homogenous muscle tissue was estimated. To do this, the sample ROI was taken in a homogenous LM muscle region which was clean of fat (Fig 2B). From this muscle homogenous region the total muscle fat index (MFI) was calculated according to the following formula: “SI_fat_/(SI_fat_ +SI_water_)” [59].

To eliminate individual differences, all estimated CSAs and MFI ROIs were normalized to the vertebral body at UL L4 using the following formula: CSA/vertebrae body area [27,28]. The intraclass correlation coefficient for intra-rater reliability was excellent for both total CSA (ICC = 0.91) and lean CSA (ICC = 0.87) of the lumbar multifidus, calculated from measurements of 5 subjects.

#### Muscle functional measurement.

The muscle functional magnetic resonance imaging (mfMRI) is a non-invasive method that measures exercise-related muscle activation by evaluating the amount of metabolic activity based on the quantitative imaging of changes in T2-relaxation times with contraction. This technique is a valid tool for assessing lumbar back muscle activity with excellent intra- and inter-rater agreement [60,61].

On the sagittal localizing scan, three transversal slices were positioned equally at the angle of vertebral inclination of the level that corresponds with UL L3, UL L4 and lower level (LL) L4. A spin-echo multi-contrast sequence was used to capture the T2-weighted images in the presence of the following parameters: repetition time 1000ms, echo train of 16 echoes ranging from 10 to 162ms, acquisition matrix*176mm^2^, field of view (FOV) 340 mm, voxel size 1.3*1.3*5.0 mm3 and total scan-time of 5min52s. The T2-weighted images were obtained twice, once after 50 minutes of lying down in rest position (T2-rest) and once immediately after completing the exercise described in 2.5.3 (T2-exercise). The T2-rest resembles muscle tissue characteristics. Existing literature suggests that lower T2-rest values indicate a higher portion of glycolytic fibers, whereas a higher resting state T2-value is related to more oxidative fibers [23,62].

A T2-Processor, 2016 software (copyright P. Vandemaele, Eng. m GIFMI UZGent) that has been implemented in the Matlab software environment (MathWork, Inc., 2016) was used to convert the mfMRI images into T2-maps to calculate the mean transverse relaxation times of the muscle tissue. In all 48 sequenced images, a T2-value per voxel (in ms) was calculated out of 15 echoes for each slice. The first 16 echoes were excluded for better curve fitting. Subsequently, the ROIs were manually and bilaterally traced on the T2-maps for the LM along the muscular borders, thus avoiding visual fat, connective tissues, or blood vessels (Fig 3) [23]. Finally, for each ROI, the mean T2-value was calculated [63]. The researcher who processed the mfMRI data was blinded to the participant characteristics.

To assess changes in the relaxation time of muscle water (T2-relaxation time) which resulted of the muscle work performed during the exercise, the shifts in T2-relaxation times were quantified and expressed as the T2-shift [64]. The T2-shift is calculated according to the following formula: ((T2-exercise - T2-rest)/T2-rest)*100. A higher T2-shift implies enhanced levels of (metabolic) activity within the LM muscle when performing the exercise, which is the result of more activity of this muscle.

The intraclass-correlation coefficient for the intra-rater agreement of the T2-values for the LM taken at 5 different subjects was 0.92, indicating a good to excellent reliability.

#### Exercise protocol.

A standardized dynamic-static trunk extension exercise of low load intensity was performed to activate and measure the metabolic activity of the LM muscle [60,65].

To perform the trunk extension exercises participants were installed in prone position on a variable-angle chair, with their upper body in 45° of trunk flexion. The anterior superior iliac spine (ASIS) was positioned on the edge of the table and the ankles strapped (for fixation) to the table. The participants placed their hands on the opposite shoulders. During the dynamic–static trunk extension exercise, the trunk was raised or extended from 45° trunk flexion to the horizontal in 2s (Fig 4A), maintained the horizontal position during 5s (Fig 4B), and returned to the start position of 45° trunk flexion in 2s. To ensure a correct horizontal position, the participants were provided with tactile feedback. A metronome (60 beats/minute) was used to ensure appropriate timing of the different movements.

A set of 10 repetitions at an exercise intensity of 40% one-repetition maximum (1-RM) was calculated, using Holten’s diagram which has been extensively reported in the literature [23,61]. The individual 1-RM was indirectly determined by registering the maximum number repetitions participants could perform with the weight of their upper body as the exercise weight during the pre-screening session. The 1-RM test was executed in the same position, over the same range of motion, and with an identical timing as during the respective exercise modality. Afterwards, the exercise load (kg) corresponding to 40% of 1-RM, was estimated using the Holten diagram which describes the relation between the performed number of repetitions and the exercise intensity [66]. The individual exercise weight was calculated using the following formula: upper body weight (kg)*exercise load (40% 1-RM)/exercise load determined (Holten diagram) [62,67]. The weight of the upper body is calculated as 70% of the total body weight. To adjust the exercise weight during the experimental session, the body was assisted via a load pulley system or extra weights were added to the trunk by holding weight pockets against the chest by crossing their arms.

### Statistical analysis

The statistical analyses were carried out using SPSS Version 26.0 (IBM, Armonk, New York) and the statistical significance level was set at p < .05. Descriptives ((mean, median, standard deviation (SD), and range (minimum to maximum)) were calculated and normality of the data was assessed using the Shapiro-Wilk’s test and visual assessment of the histograms, boxplots and QQ-plots. Depending on the data distribution, comparability of the groups in regards to BMI, demographic and disorder related characteristics was examined using the independent-samples T or Mann-Whitney U test.

A linear mixed model was used to examine the relationship between LM muscle structure and function and brain structural characteristics, while considering the group (LBP versus HC (HC is the reference group)) as a fixed factor. The GMT and GMV of the cortices as the outcome measures. Age, BMI, pain intensity, pain duration, HADS, PCS, SF-36 and RMDQ were selected as covariates because these factors have been shown to influence the muscle structure/function [28,68–75] as well as the brain structure [12,76–79]. The linear mixed model was built using a backward stepwise technique as full model. These backward stepwise model includes all variables and deleted one by one from the full model until all remaining variables are considered to have some significant contribution to the outcome [80]. The linear mixed models were built for correlated measures by including the muscle and brain as main effects, group as constant factors and covariates as well as a random intercept for the study participants. Pairwise post-hoc Bonferroni adjusted comparisons were performed in case of a significant group effect. A priori power caculations (By Gpower) was conducted and indicated 24 subjects was needed to reach a power of 0.80. All models were individually checked via the effect of parameter estimates, standard error, t-value, and 95% confidence interval.

## Results

### Demographic variables

The patients with NSLBP were older (29.3 ± 7.9, p = 0.001), had a higher BMI (24.0 ± 3.2, p = 0.01), a higher score for depression on the HADS (3.5 ± 4.4, p = 0.01), a higher score on the PCS (15.9 ± 8.2, p = 0.03), low quality of life on the SF-36 (619.1 ± 78.6, p = 0.01) and disability on the RMDQ (4.2 ± 3.7, p = 0.01) compared to HC. A borderline significant difference between the groups was found for HADS anxiety showing a higher HADS anxiety in patients with LBP (6.4 ± 4.6, p = 0.052). All the demographic variables are presented in Table 1.

**Table 1:** Participants baseline demographic characteristics.

			Mean	Median	SD	Range	P-value
**Demographic characteristics**	**Age** **(years)** ^***b***^	***HC***	22.35	22.00	1.33	19.00 −25.00	0.001*
		***LBP***	29.26	27.00	7.99	19.0-45.0	
	**BMI** **(kg/m** ^**2**^ **)** ^***b***^	***HC***	21.72	21.87	2.11	18.00 - 25.65	0.013*
		***LBP***	23.94	23.42	3.21	19.61-29.10	
**LBP characteristics**	**Current LBP intensity (NRS/10)** ^***b***^	***HC***	0.00	0.00	0.00	0.00 −.00	0.001*
		***LBP***	2.80	4.00	2.62	0.00 - 6.00	
	**LBP duration** **(months)** ^***a***^	***HC***	0.00	0.00	0.00	0.00-0.00	0.001*
		***LBP***	89.1	60.0	66.9	3.0-204.0	
**Psychological characteristics**	**Anxiety** **(HADSA/21)** ^***a***^	***HC***	4.00	4.00	2.07	0.00-8.00	0.052
		***LBP***	6.40	6.00	4.67	1.00-19.00	
	**Depression** **(HADSD/21)** ^***a***^	***HC***	0.50	0.00	0.65	0.00-2.00	0.001*
		***LBP***	3.46	2.00	4.33	0.0-16.0	
	**Pain catastrophizing (PCS)** ^***b***^	***HC***	10.92	10.50	8.19	0.00-22.00	0.027*
		***LBP***	15.93	16.00	8.36	2.00-30.00	
**Health status**	**SF-36** **(Total Score)** ^***b***^	***HC***	690.8	715.8	63.7	57.5 - 768.0	0.012*
		***LBP***	619.10	650.50	78.60	463.00 - 730.00	
**LBP disability**	**RMDQ**	***HC***	0.00	0.00	0.00	0.00 - 0.00	0.001*
		***LBP***	4.20	3.00	3.70	0.00 −12.00	
**LM macrostructure**	**Total CSA** ^***a***^	***HC***	5.37	5.5	.98	3.93 - 7.67	0.73
		***LBP***	4.56	4.5	1.32	2.62 - 7.42	
	**Fat CSA** ^***b***^	***HC***	.66	.60	.23	.34 −1.24	.425
		***LBP***	.64	.53	.37	.28 −1.73	
	**Muscle CSA** ^***a***^	***HC***	4.70	4.69	.78	3.50–6.44	0.047*
		***LBP***	3.92	3.73	1.20	2.34–6.57	
**LM fibers tissue characteristics**	**T2-rest UL L3** ^***b***^	***HC***	44.34	43.66	2.65	40.45–49.09	0.059
		***LBP***	46.92	46.14	4.23	41.29–56.96	
	**T2-rest UL L4** ^***b***^	***HC***	44.67	44.65	1.92	40.55–48.01	.145
		***LBP***	43.68	44.11	1.58	40.60–46.51	
	**T2-rest LL L4** ^***b***^	***HC***	45.23	45.22	2.00	42.26–49.58	.001**
		***LBP***	42.65	44.11	1.75	39.66–45.51	
**LM lean muscle tissue**	**MFI %** ^***b***^	***HC***	6.96	6.41	1.86	5.33-12.59	0.050*
		***LBP***	8.58	7.50	3.25	5.91-16.80	
**LM metabolic activity**	**T2-shift UL L3** ^***a***^	***HC***	3.14	2.95	2.80	−3.15–9.83	.914
		***LBP***	3.87	3.12	3.08	.01–10.13	
	**T2-shift UL L4** ^***b***^	***HC***	2.71	3.11	2.00	−1.62–6.52	.427
		***LBP***	3.22	3.25	1.27	.56–5.02	
	**T2-shift LL L4** ^***a***^	***HC***	4.11	3.35	2.74	.85–9.24	.983
		***LBP***	3.93	3.29	2.60	1.53–10.65	

Legend: The distribution of the continuous variables within each group was assessed by histogram, QQ-plots and the Shapiro-Wilk test. ^a^ = Data which were not normally distributed and subsequent group differences were analysed using the Mann-Whitney U test, ^*b*^ = Data which were assumed to be normally distributed and analysed with independent t-test, LBP = low back pain, HC = healthy control, BMI = body mass index, NRS = numeric rating scale, HADSA = hospital anxiety depression scale for anxiety, HADSD = hospital anxiety depression scale for depression, PCST = pain catastrophizing scale total score, SF-36 = Short form, RMDQ = Rolland Moris Disability Questionnaire, LM = Lumbar multifidus, CSA = cross sectional area, UL L = Upper Level of Lumbar, LL L = Lower Level of lumbar, MFI = muscle fat index, % = percentage, ** = p < .001, * = p < .005.

### Association between CSA, fat CSA and MFI of the LM muscle, and GMT and GMV of the cortices

According to the results presented in Table 2, no significant associations were found between LM CSA, and GMT or GMV of any examined sensorimotor cortices (p ≥ .05).

**Table 2:** Model characteristics and parameter estimates using linear mixed models for associations between muscle CSA of LM, GMT and GMV of sensorimotor cortices.

	MODEL		PARAMETER ESTIMATES		SE	T	p-value
***MUSCLE CSA***	***GMT***	**Rostral middle frontal (mm)**	Intercept	2.22	0.09	24.88	0.001
			Group	−0.02	0.04	−0.38	0.704
			Muscle CSA LM	0.04	0.00	1.27	0.209
		**Caudal middle frontal (mm)**	Intercept	2.55	0.09	28.21	0.001
			Group	0.04	0.05	25.96	0.443
			Muscle CSA LM	0.00	0.00	−0.02	0.985
		**Precentral (mm)**	Intercept	1.74	0.28	6.11	0.001
			Group	0.05	0.05	0.91	0.372
			Muscle CSA LM	0.00	0.00	−1.24	0.224
			***Current LBP***	0.03	0.00	2.14	0.044 *
			***PCS***	0.01	0.01	2.39	0.026 *
			***SF-36***	0.00	0.00	2.24	0.003 **
		**Postcentral (mm)**	Intercept	1.97	0.08	27.35	0.001
			Group	0.01	0.04	0.25	0.806
			Muscle CSA LM	0.00	0.00	0.92	0.362
			***HADSA***	0.02	0.01	2.97	0.007 **
		**Insula(mm)**	Intercept	2.65	0.25	10.75	0.001
			Group	−0.15	0.07	−2.03	0.054
			Muscle CSA LM	0.00	0.00	−0.13	0.267
			**Pain duration**	0.00	0.00	−2.19	0.039 *
			**HASDD**	0.03	0.01	2.08	0.049 *
			**SF-36**	0.00	0.00	2.77	0.011 *
		**Superior parietal (mm)**	Intercept	2.21	0.07	49.86	0.001
			Group	−0.03	0.04	−0.60	0.555
			Muscle CSA LM	0.00	0.00	0.32	0.752
		**Supramarginal (mm)**	Intercept	2.67	0.09	28.49	0.001
			Group	0.05	0.05	1.08	0.288
			Muscle CSA LM	0.00	0.00	−0.27	0.792
		**Paracentral (mm)**	Intercept	2.46	0.08	29.64	0.001
			Group	0.03	0.05	0.60	0.557
			Muscle CSA LM	0.00	0.00	0.10	0.92
		**Posterior cingulate (mm)**	Intercept	2.51	0.09	27.61	0.001
			Group	−0.07	0.05	−1.59	0.123
			Muscle CSA LM	0.01	0.00	1.82	0.078
		**Rostral anterior cingulate (mm)**	Intercept	2.81	0.15	19.29	0.001
			Group	0.01	0.07	0.13	0.894
			Muscle CSA LM	0.00	0.00	0.99	0.327
			***HADSD***	0.03	0.02	2.30	0.025 *
	***GMV***	**Rostral middle frontal (mm** ^**3**^ **)**	Intercept	16763.57	1443.02	11.62	0.001
			Group	294. 559	825.81	0.36	0.724
			Muscle CSA LM	−34.83	46.66	−0.75	0.459
		**Caudal middle frontal (mm** ^**3**^ **)**	Intercept	7520.91	1173.63	6.41	0.001
			Group	−154.54	663.03	−0.23	0.818
			Muscle CSA LM	−12.21	31.70	−0.39	0.702
			***Pain duration***	−11.15	5.16	−2.16	0.041 *
				**Precentral (mm** ^**3**^ **)**	Intercept	12898.13	1089.61	11.84	0.001
					Group	136.24	625.61	0.22	0.829
					Muscle CSA LM	19.47	35.21	0.55	0.583
		**Postcentral (mm** ^**3**^ **)**	Intercept	9235.45	926.83	9.97	0.001
			Group	−109.49	491.20	−0.22	0.825
			Muscle CSA LM	15.80	30.38	0.52	0.606
		**Insula (mm** ^**3**^ **)**	Intercept	6743.65	525.05	12.84	0.001
			Group	−31.67	347.13	−0.09	0.928
			Muscle CSA LM	4.28	16.41	0.26	0.795
		**Superior parietal (mm** ^**3**^ **)**	Intercept	13306.54	908.85	14.64	0.001
			Group	42.04	496.16	0.09	0.933
			Muscle CSA LM	−11.87	29.64	−0.40	−0.4
		**Supramarginal (mm** ^**3**^ **)**	Intercept	2551.51	2427.89	1.05	0.304
			Group	−75.41	465.99	−0.16	0.873
			Muscle CSA LM	−10.88	30.94	−0.35	0.727
			***PCS***	70.00	28.84	2.43	0.023 *
			***SF-36***	11.58	3.47	3.34	0.003 *
		**Paracentral (mm** ^**3**^ **)**	Intercept	3372.36	527.09	6.40	0.001
			Group	256.39	275.28	0.93	0.36
			Muscle CSA LM	9.63	17.32	0.56	0.581
		**Posterior cingulate (mm** ^**3**^ **)**	Intercept	3333.74	321.64	10.37	0.001
			Group	52.47	163.23	0.32	0.75
			Muscle CSA LM	−0.70	10.61	−0.07	0.948
**Rostral anterior cingulate (mm** ^**3**^ **)**	Intercept	2315.72	342.06	6.77	0.001
	Group	61.17	168.61	0.36	0.718
	Muscle CSA LM	6.25	11.34	0.55	0.584

*p < .05, ** p < .01.

Legend: SE: standard error; t: t-value; CI: confidence interval), GMV: grey matter volume, LM: lumbar multifidus, MFI: muscle fat index. SF-36: Medical Outcomes Study 36-item short form health survey, HADSA: Hospital Anxiety Depression Scale for Anxiety, HADSD: Hospital Anxiety Depression Scale for Depression, PCS: pain catastrophizing scale.

Results in Table 3 also showed no significant associations between the LM fat CSA, and GMT or GMV of any cortices (p ≥ .05).

**Table 3:** Model characteristics and parameter estimates using linear mixed models for associations between fat CSA of LM, GMT and GMV of sensorimotor cortices.

	MODEL		PARAMETER ESTIMATES		SE	T	p-value
***FAT CSA***	***GMT***	**Rostral middle frontal (mm)**	Intercept	2.37	0.05	47.47	0.001
			Group	0.01	0.04	0.16	0.872
			Fat CSA LM	−0.01	0.01	−0.93	0.359
		**Caudal middle frontal (mm)**	Intercept	2.56	0.05	50.88	0.001
			Group	0.04	0.04	0.84	0.408
			Fat CSA LM	0.00	0.01	−0.24	0.812
		**Precentral (mm)**	Intercept	2.07	0.23	8.98	0.001
			Group	0.00	0.05	0.09	0.930
			Fat CSA LM	0.00	0.01	0.23	0.823
			***HADSD***	0.02	0.01	2.10	0.046 *
			***SF-36***	0.00	0.00	2.41	0.024 *
		**Postcentral (mm)**	Intercept	2.04	0.05	38.20	0.001
			Group	0.02	0.04	0.56	0.583
			Fat CSA LM	0.00	0.01	−0.01	0.993
			***HADSA***	0.02	0.01	2.83	0.009 *
		**Insula(mm)**	Intercept	3.24	0.06	57.90	0.001
			Group	−0.04	0.05	−0.74	0.468
			Fat CSA LM	−0.01	0.01	−1.21	0.231
		**Superior parietal (mm)**	Intercept	2.26	0.04	54.37	0.001
			Group	−0.02	0.04	−0.05	0.608
			Fat CSA LM	−0.01	0.01	−1.22	0.227
		**Supramarginal (mm)**	Intercept	2.71	0.05	52.21	0.001
			Group	0.05	0.05	1.10	0.284
			Fat CSA LM	−0.01	0.01	−1.57	0.125
		**Paracentral (mm)**	Intercept	2.51	0.05	54.67	0.001
			Group	0.03	0.04	0.71	0.483
			Fat CSA LM	−0.01	0.01	−1.25	0.216
		**Posterior cingulate (mm)**	Intercept	2.29	0.20	11.73	0.001
			Group	−0.08	0.05	−1.78	0.087
			Fat CSA LM	−0.01	0.01	−0.70	0.492
			**SF-36**	0.01	0.00	2.15	0.042 *
		**Rostral anterior cingulate (mm)**	Intercept	3.12	0.07	42.23	0.001
			Group	−0.03	0.06	−0.56	0.577
			Fat CSA LM	−0.02	0.01	−1.56	0.125
	***GMV***	**Rostral middle frontal (mm** ^**3**^ **)**	Intercept	16274.91	815.13	19.97	0.001
			Group	101.61	775.78	0.13	0.897
			Fat CSA LM	−114.62	136.26	−0.84	0.404
		**Caudal middle frontal (mm** ^**3**^ **)**	Intercept	7532.70	720.46	10.46	0.001
			Group	−188.72	665.20	−0.28	0.779
			Fat CSA LM	−87.83	92.59	−0.95	0.348
			***Pain duration***	−10.86	5.06	−2.15	0.042 *
		**Precentral (mm** ^**3**^ **)**	Intercept	13692.19	612.63	22.35	0.001
			Group	258.38	580.68	0.45	0.660
			Fat CSA LM	−53.66	102.75	−0.52	0.604
		**Postcentral (mm** ^**3**^ **)**	Intercept	9499.08	515.24	18.44	0.001
			Group	−20.81	454.96	−0.05	0.956
			Fat CSA LM	42.52	90.95	0.47	0.642
		**Insula (mm** ^**3**^ **)**	Intercept	7208.96	305.09	23.63	0.001
			Group	3.17	325.94	0.01	0.992
			Fat CSA LM	−77.49	45.18	−1.72	0.093
		**Superior parietal (mm** ^**3**^ **)**	Intercept	12700.71	507.58	25.02	0.001
			Group	−35.78	462.60	−0.08	0.939
			Fat CSA LM	60.20	87.71	0.69	0.496
		**Supramarginal (mm** ^**3**^ **)**	Intercept	423.71	2586.42	0.16	0.871
			Group	36.37	429.73	0.09	0.933
			Fat CSA LM	−14.74	89.43	−0.17	0.870
			***PCS***	76.74	28.47	2.70	0.013 *
			***HADSA***	157.71	75.71	2.08	0.049 *
			***SF-36***	13.03	3.32	3.93	0.001 **
		**Paracentral (mm** ^**3**^ **)**	Intercept	3714.13	291.20	12.76	0.001
			Group	315.43	252.56	1.25	0.223
			Fat CSA LM	−14.98	51.97	41.51	0.776
		**Posterior cingulate (mm** ^**3**^ **)**	Intercept	3256.13	177.87	18.31	0.001
			Group	46.74	149.97	0.31	0.758
			Fat CSA LM	13.02	32.27	0.40	0.689
		**Rostral anterior cingulate (mm** ^**3**^ **)**	Intercept	2431.51	189.95	12.80	0.001
			Group	96.56	154.85	0.62	0.536
			Fat CSA LM	14.21	35.08	0.41	0.687

*p < .05, ** p < .01.

Legend: SE: standard error; t: t-value; CI: confidence interval, GMV: grey matter volume, LM: lumbar multifidus, CSA: cross sectional area. SF-36: Medical Outcomes Study 36-item short form health survey, HADSA: Hospital Anxiety Depression Scale for Anxiety, HADSD: Hospital Anxiety Depression Scale for Depression, PCS: pain catastrophizing scale.

Results of the association between LM MFI, and GMT or GMV of the sensorimotor cortices are presented in Table 4. The LM MFI was found to be correlated with the GMV of two cortices. More specifically, a significant positive correlation was found between the MFI and the GMV of the paracentral (p = 0.03,) and the postcentral cortex (p = 0.05,). However, none of the LM MFI and GMV associates were affected by group effects (p > .05). Lastly, there were no significant correlations between LM MFI and the GMT or GMV of frontal, middle, posterior and cingulate cortices (p > .05).

**Table 4:** Model characteristics and parameter estimates using linear mixed models for associations between muscle fat index (MFI) of LM, GMT and GMV of sensorimotor cortices.

	MODEL		PARAMETER ESTIMATES		SE	T	p-value
***MFI LM***	***GMT***	**Rostral middle frontal (mm)**	Intercept	2.28	0.06	38.18	0.001
			Group	0.01	0.04	0.32	0.75
			MFI LM	0.01	0.01	0.93	0.359
		**Caudal middle frontal (mm)**	Intercept	2.48	0.06	43.96	0.001
			Group	0.05	0.04	1.07	0.294
			MFI LM	0.01	0.01	1.39	0.169
		**Precentral (mm)**	Intercept	2.07	0.22	9.60	0.001
			Group	0.01	0.05	0.17	0.87
			MFI LM	0.00	0.00	0.55	0.583
			***HADSD***	0.02	0.01	2.22	0.037*
			***SF-36***	0.01	0.00	2.44	0.023 *
		**Postcentral (mm)**	Intercept	2.02	0.05	40.48	0.001
			Group	0.02	0.04	0.65	0.525
			MFI LM	0.00	0.00	0.89	0.381
			***HADSA***	0.02	0.01	2.76	0.011 *
		**Insula(mm)**	Intercept	2.55	0.26	9.96	0.001
			Group	−0.15	0.08	−1.99	0.059
			MFI LM	0.01	0.01	1.50	0.141
			***Pain duration***	0.00	0.00	−2.19	0.038 *
			***HADSD***	0.03	0.01	2.18	0.040*
			***SF-36***	0.00	0.00	2.53	0.019 *
		**Superior parietal (mm)**	Intercept	2.22	0.04	57.23	0.001
			Group	−0.02	0.04	−0.50	0.62
			MFI LM	0.00	0.00	0.31	0.758
		**Supramarginal (mm)**	Intercept	2.70	0.05	50.95	0.001
			Group	0.04	0.05	0.86	0.399
			MFI LM	−0.01	0.01	−1.29	0.204
		**Paracentral (mm)**	Intercept	2.44	0.04	55.81	0.001
			Group	0.03	0.04	0.81	0.427
			MFI LM	0.00	0.00	1.06	0.297
		**Posterior cingulate (mm)**	Intercept	2.28	0.19	12.11	0.001
			Group	−0.09	0.05	−1.94	0.064
			MFI LM	0.00	0.01	−0.61	0.548
			***SF-36***	0.01	0.00	2.26	0.033 *
		**Rostral anterior cingulate (mm)**	Intercept	3.00	0.10	30.15	0.001
			Group	0.02	0.07	0.28	0.777
			MFI LM	−0.01	0.01	−0.60	0.533
			***HADSD***	0.03	0.01	2.05	0.046 *
	***GMV***	**Rostral middle frontal (mm** ^**3**^ **)**	Intercept	1504.73	757.69	19.86	0.001
			Group	202.17	788.46	0.26	0.800
			MFI LM	90.00	64.56	1.39	0.172
		**Caudal middle frontal (mm** ^**3**^ **)**	Intercept	6627.54	541.36	12.24	0.001
			Group	746.90	490.02	1.52	0.139
			MFI LM	−63.90	52.33	−1.22	0.228
		**Precentral (mm** ^**3**^ **)**	Intercept	13466.56	576.16	23.37	0.001
			Group	249.97	588.66	0.43	0.675
			MFI LM	−1.48	50.12	−0.03	0.977
		***Postcentral (mm***^***3***^)	Intercept	8885.78	497.51	17.86	0.001
			Group	111.81	436.51	0.26	0.800
			***MFI LM***	100.20	49.09	2.04	0.047 *
		**Insula (mm** ^**3**^ **)**	Intercept	6941.54	282.46	24.58	0.001
			Group	−18.26	331.28	−0.06	0.956
			MFI LM	−9.44	19.86	−0.48	0.638
		**Superior parietal (mm** ^**3**^ **)**	Intercept	13363.79	487.99	27.39	0.001
			Group	−91.54	466.47	−0.20	0.846
			MFI LM	−49.59	45.25	−1.10	0.279
		**Supramarginal (mm** ^**3**^ **)**	Intercept	2393.31	2321.26	1.03	0.314
			Group	−79.20	447.94	−0.18	0.861
			MFI LM	26.15	59.77	0.44	0.664
			***PCS***	68.41	28.65	2.39	0.025 *
			***SF-36***	11.04	3.35	3.30	0.023 *
		***Paracentral (mm***^***3***^)	Intercept	3135.93	288.94	10.85	0.001
			Group	395.01	252.68	1.59	0.130
			***MFI LM***	64.00	28.57	2.24	0.030 *
		**Posterior cingulate (mm** ^**3**^ **)**	Intercept	3298.54	193.78	17.02	0.001
			Group	50.74	151.44	0.34	0.740
			MFI LM	1.90	20.32	0.09	0.926
		**Rostral anterior cingulate (mm** ^**3**^ **)**	Intercept	2547.76	227.78	11.19	0.001
			Group	89.80	158.15	0.57	0.573
			MFI LM	−6.67	24.97	−0.27	0.790

*p < .05, ** p < .01.

Legend: SE: standard error; t: t-value; CI: confidence interval, GMV: grey matter volume, LM: lumbar multifidus, CSA: cross sectional area. SF-36: Medical Outcomes Study 36-item short form health survey, HADSA: Hospital Anxiety Depression Scale for Anxiety, HADSD: Hospital Anxiety Depression Scale for Depression, PCS: pain catastrophizing scale.

### Association between the T2-rest of the LM muscle and GMT, GMV of the cortices

Results of the association between T2-rest of the LM and GMT and GMV of the sensorimotor cortices are presented in Table 5. The T2-rest of the LM was found to be correlated with the GMT of the frontal cortical regions. More specifically, a significant positive correlation was found between T2-rest of the LM at UL L3 (p = 0.020) and at UL L4 (p = 0.05), and the GMT of the rostral middle frontal cortex. However, the factor group did not show to have any effect on these correlations (p > .05). No associations were recorded between the T2-rest of LM at LL L4 and the rostral middle frontal GMT (p > .05).

**Table 5:** Model characteristics and parameter estimates using linear mixed models for associations between T2-rest of LM, GMT and GMV of sensorimotor cortices.

	MODEL		PARAMETER ESTIMATES		SE	T	p-value
***T2-REST***	***GMT***	**Rostral middle frontal (mm)**	Intercept	1.77	0.22	7.57	0.001
			Group	0.03	0.04	0.65	0.517
			***UL L3 LM***	0.01	0.01	2.41	0.020 *
			Intercept	1.67	0.32	5.15	0.001
			Group	0.00	0.40	0.01	0.990
			***UL L4 LM***	0.02	0.01	2.04	0.046 *
			Intercept	1.81	0.40	4.48	0.001
			Group	0.02	0.41	0.37	0.711
			LL L4 LM	0.01	0.01	1.31	0.197
		**Caudal middle frontal (mm)**	Intercept	1.89	0.23	8.30	0.001
			Group	0.06	0.04	1.47	0.153
			***UL L3 LM***	0.01	0.01	2.89	0.007 *
			Intercept	1.68	0.30	5.55	0.001
			Group	0.03	0.04	0.75	0.458
			***UL L4 LM***	0.02	0.01	2.90	0.007 **
			Intercept	1.47	0.36	4.11	0.001
			***Group***	0.10	0.04	2.38	0.025 *
			***LL L4 LM***	0.02	0.01	2.90	0.006 **
			***HADSD***	0.02	0.01	2.39	0.025 *
		**Precentral (mm)**	Intercept	1.89	0.22	8.64	0.001
			Group	0.03	0.05	0.72	0.480
			***UL L3 LM***	0.02	0.00	3.35	0.001 **
			Intercept	1.42	0.38	3.71	0.001
			Group	0.00	0.05	−0.02	0.986
			***UL L4 LM***	0.02	0.01	2.15	0.037 *
			***HADSD***	0.03	0.11	2.28	0.032 *
			***SF-36***	0.00	0.00	2.52	0.019 *
			Intercept	1.31	0.42	3.14	0.003
			Group	0.02	0.05	0.37	0.716
			***LL L4 LM***	0.02	0.01	2.15	0.036 *
			***HADSD***	0.03	0.01	2.57	0.016 *
			***SF-36***	0.00	0.00	2.82	0.009 **
		**Postcentral (mm)**	Intercept	1.85	0.19	9.66	0.000
			Group	0.00	0.04	0.05	0.957
			***UL L3 LM***	0.01	0.00	1.57	0.124
			Intercept	1.75	0.27	6.61	0.000
			Group	−0.01	0.04	−0.29	0.778
			***UL L4 LM***	0.01	0.01	1.48	0.146
			Intercept	1.94	0.27	7.16	0.000
			Group	−0.01	0.04	−0.12	0.908
			***LL L4 LM***	0.01	0.01	0.77	0.446
		**Insula (mm)**	Intercept	3.16	0.26	12.05	0.001
			Group	−0.04	0.05	−0.72	0.479
			UL L3 LM	0.00	0.01	0.09	0.930
			Intercept	3.03	0.33	8.36	0.001
			Group	−0.04	0.05	−0.77	0.448
			UL L4 LM	0.00	0.01	0.43	0.664
			Intercept	3.31	0.38	8.78	0.001
			Group	−0.04	0.05	−0.79	0.440
			LL L4 LM	0.00	0.01	−0.33	0.740
		**Superior parietal (mm)**	Intercept	1.73	0.17	10.35	0.001
			Group	0.02	0.00	−0.10	0.925
			***UL L3 LM***	0.11	0.00	3.04	0.004 **
			Intercept	1.83	0.25	7.41	0.001
			Group	−0.03	0.04	−0.60	0.522
			UL L4 LM	0.01	0.01	1.63	0.110
			Intercept	2.07	0.24	8.59	0.001
			Group	−0.02	0.04	−0.46	0.649
			LL L4 LM	0.00	0.01	0.67	0.508
		**Supramarginal (mm)**	Intercept	2.64	0.25	10.69	0.001
			Group	0.05	0.05	1.05	0.306
			UL L3 LM	0.00	0.01	0.02	0.983
			Intercept	2.15	0.33	6.48	0.001
			Group	0.04	0.04	1.00	0.325
			UL L4 LM	0.01	0.01	1.51	0.138
			Intercept	2.37	0.37	6.40	0.001
			Group	0.05	0.04	1.19	0.245
			LL L4 LM	0.01	0.01	0.74	0.465
		**Paracentral (mm)**	Intercept	2.61	0.21	12.39	0.001
			Group	0.02	0.05	0.53	0.601
			UL L3 LM	0.00	0.00	−0.70	0.490
			Intercept	2.44	0.29	8.35	0.001
			Group	0.03	0.04	0.68	0.511
			UL L4 LM	0.01	0.01	0.10	0.924
			Intercept	2.49	0.30	8.43	0.001
			Group	0.03	0.04	0.66	0.515
			LL L4 LM	−0.01	0.01	−0.07	0.942
		**Posterior cingulate (mm)**	Intercept	1.95	0.24	8.04	0.001
			Group	−0.01	0.04	−0.32	0.751
			***UL L3 LM***	0.02	0.00	2.98	0.005 **
			Intercept	1.37	0.36	3.85	0.001
			***Group***	−0.01	0.05	−2.66	0.014 *
			***UL L4 LM***	0.02	0.01	2.43	0.022 *
			***HADSD***	0.03	0.01	3.06	0.006 **
			***SF-36***	0.00	0.00	3.35	0.0013 *
			***RMDQ***	−0.02	0.01	−2.65	0.015 *
			Intercept	1.09	0.46	2.36	0.024
			Group	−0.09	0.05	−1.86	0.076
			***LL L4 LM***	0.02	0.01	2.45	0.019 *
			***HADSD***	0.03	0.01	2.44	0.007 **
			***SF-36***	0.00	0.00	2.93	0.003 **
			***RMDQ***	−0.02	0.01	−2.19	0.039 *
	***GMV***	**Rostral anterior cingulate (mm** ^**3**^ **)**	Intercept	2.55	0.35	7.19	0.001
			Group	0.04	0.07	0.60	0.548
			UL L3 LM	0.01	0.01	1.14	0.259
			***HADSD***	0.03	0.01	2.04	0.047 *
			Intercept	2.52	0.49	5.12	0.001
			Group	0.03	0.07	0.41	0.683
			UL L4 LM	0.01	0.01	0.88	0.382
			***HADSD***	0.03	0.01	2.19	0.033 *
			Intercept	2.64	0.61	4.33	0.001
			Group	0.04	0.07	0.54	0.592
			***LL L4 LM***	0.03	0.02	2.18	0.034 *
		**Rostral middle frontal (mm** ^**3**^ **)**	Intercept	17238.85	3640.43	4.74	0.001
			Group	34.00	785.67	0.04	0.966
			UL L3 LM	−31.56	77.20	−0.41	0.684
			Intercept	12852.36	5051.55	2.54	0.014
			Group	65.16	777.33	0.08	0.934
			UL L4 LM	68.73	118.39	0.58	0.564
			Intercept	5843.98	4802.05	1.22	0.230
			Group	271.29	775.64	0.35	0.729
			***LL L4 LM***	233.79	112.40	2.08	0.043 *
		**Caudal middle frontal (mm** ^**3**^ **)**	Intercept	2109.44	2567.93	0.82	0.416
			Group	974.38	512.75	1.90	0.069
			UL L3 LM	85.95	54.55	1.58	0.122
			Intercept	96.35	3286.20	0.03	0.977
			Group	−223.13	656.91	−0.34	0.737
			***UL L4 LM***	163.91	77.74	2.11	0.041 *
			***Pain duration***	−10.54	4.99	−2.11	0.045 *
			Intercept	−337.01	3692.55	−0.09	0.928
			Group	947.58	477.95	1.98	0.058
			LL L4 LM	152.04	86.63	1.76	0.085
		**Precentral (mm** ^**3**^ **)**	Intercept	9776.05	2698.82	3.62	0.001
			Group	386.08	591.13	0.65	0.519
			UL L3 LM	78.90	57.21	1.38	0.174
			Intercept	13601.09	3813.91	3.57	0.001
			Group	252.99	590.54	0.43	0.672
			UL L4 LM	−3.45	89.37	−0.04	0.969
			Intercept	12665.53	3780.58	3.35	0.002
			Group	266.46	589.74	0.45	0.655
			LL L4 LM	18.59	88.53	0.21	0.210
		**Postcentral (mm** ^**3**^ **)**	Intercept	4299.53	2336.81	1.84	0.072
			Group	180.93	456.21	0.40	0.695
			***UL L3 LM***	115.56	49.66	2.33	0.024 *
			Intercept	1468.58	3072.88	0.48	0.635
			Group	−79.13	422.01	−0.19	0.853
			***UL L4 LM***	193.75	73.11	2.65	0.011 *
			Intercept	−1504.54	3246.08	−0.46	0.645
			Group	191.43	398.35	0.48	0.635
			***LL L4 LM***	263.67	76.19	3.46	0.001 **
		**Insula (mm** ^**3**^ **)**	Intercept	7867.42	1229.33	6.40	0.001
			Group	−42.79	341.04	−0.13	0.901
			UL L3 LM	−21.48	25.86	−0.83	0.410
			Intercept	8207.11	1718.95	4.77	0.001
			Group	4.11	341.38	0.01	0.990
			UL L4 LM	−31.62	40.12	−0.79	0.435
			Intercept	8851.84	1525.65	5.80	0.001
			Group	−42.99	341.23	−0.13	0.901
			LL L4 LM	−46.78	35.49	−1.32	0.196
		**Superior parietal (mm** ^**3**^ **)**	Intercept	11187.02	2315.39	4.83	0.001
			Group	36.48	461.29	0.08	0.938
			UL L3 LM	38.18	49.18	0.78	0.441
			Intercept	8463.91	3136.13	2.70	0.010
			Group	−63.25	439.12	−0.14	0.887
			UL L4 LM	106.16	73.57	1.44	0.156
			Intercept	9173.35	3336.42	2.75	0.008
			Group	41.73	443.47	0.09	0.926
			LL L4 LM	89.38	78.26	1.14	0.259
		**Supramarginal (mm** ^**3**^ **)**	Intercept	5635.13	2574.42	2.19	0.035
			Group	454.28	460.98	0.99	0.334
			UL L3 LM	106.36	54.79	1.94	0.060
			Intercept	−4527.89	3611.14	−1.25	0.220
			Group	−203.42	414.49	−0.49	0.628
			***UL L4 LM***	170.77	71.15	2.50	0.023 *
			***PCS***	61.38	26.42	2.32	0.029 *
			***SF-36***	11.04	3.09	3.58	0.002 **
			Intercept	−337.01	3692.55	−0.93	0.928
			Group	947.58	477.95	1.98	0.058
			LL L4 LM	152.04	86.63	1.76	0.085
		**Paracentral (mm** ^**3**^ **)**	Intercept	4555.89	1398.40	3.26	0.002
			Group	280.47	264.89	1.06	0.301
			UL L3 LM	−19.48	29.73	−0.66	0.516
			Intercept	4203.58	1917.65	2.19	0.034
			Group	317.90	257.06	1.24	0.229
			UL L4 LM	−13.10	45.01	−0.29	0.773
			Intercept	3306.35	2093.60	1.58	0.120
			Group	319.93	253.66	1.26	0.220
			LL L4 LM	8.04	49.15	0.16	0.871
		**Posterior cingulate (mm** ^**3**^ **)**	Intercept	2156.12	836.53	2.58	0.014
			Group	90.56	148.87	0.61	0.548
			UL L3 LM	24.83	17.81	1.39	0.172
			Intercept	1885.30	1144.61	1.65	0.109
			Group	37.29	145.13	0.26	0.799
			UL L4 LM	33.68	26.88	34.89	0.219
			Intercept	1330.52	1309.70	1.02	0.315
			Group	85.02	144.09	0.59	0.560
			LL L4 LM	46.72	30.76	1.52	0.136
		**Rostral anterior cingulate (mm** ^**3**^ **)**	Intercept	1291.20	821.69	1.51	0.137
			Group	−24.08	166.00	−0.15	0.885
			***UL L3 LM***	45.62	18.44	2.46	0.015 *
			***Age***	−31.72	13.17	−2.41	0.020 *
			Intercept	219.00	1164.92	0.19	0.852
			Group	−147.86	164.35	−0.90	0.372
			***UL L4 LM***	77.36	27.65	2.79	0.007 **
			***Age***	−35.13	13.15	−2.67	0.010 **
			Intercept	−1641.09	1376.20	−1.19	0.239
			Group	−83.96	157.43	−0.53	0.596
			***LL L4 LM***	125.70	32.75	3.84	0.001 **
			***Age***	−30.35	12.18	−2.49	0.016 *
			***HADSA***	−60.12	26.60	−2.26	0.028 *

* p < .05, ** p < .01.

Legend: SE: standard error; t: t-value; CI: confidence interval, LM: lumbar multifidus, UL L3: upper-level of lumbar 3; UL L4: upper-level of lumbar 4; LL L4: lower-level of lumbar 4. CSA: cross sectional area. SF-36: Medical Outcomes Study 36-item short form health survey, HADSA: Hospital Anxiety Depression Scale for Anxiety, HADSD: Hospital Anxiety Depression Scale for Depression, PCS: pain catastrophizing scale.

The T2-rest values of the LM at all assessed lumbar levels were found to be correlated with the GMT of the caudal middle frontal cortex. More specifically, a significantly positive association was found between the T2-rest of LM at UL L3 (p = 0.01), UL L4 (p = 0.01) and LL L4 (p = 0.01), and the GMT of caudal middle frontal cortex. Patients with NSLBP showed a significant higher GMT of the caudal middle frontal cortex (p = 0.025) in this association with T2-rest at LL L4 (p = 0.006) after controlling for depression (p = 0.025). However, the factor group did not affect the correlations between the T2-rest of LM at UL L3 and UL L4 and the GMT of caudal middle frontal cortex (p > .05).

Furthermore, the T2-rest of the LM was found to be correlated with the GMT of the middle cortical regions. More specifically, a significant positive correlation was found between the T2-rest of LM at UL L3 (p = 0.001) and UL L44 (p = 0.037) and LL L4 (p = 0.036), and the GMT of the precentral cortex after controlling for depression and health status. Nevertheless, the factor group did not have any effect on these correlations (p > .05). No associations were found between the T2-rest of the LM at UL L3, UL or LL L4 and the GMT of the postcentral and insular cortices (p > .05).

At the posterior cortical regions, the T2-rest value of the LM at a single lumbar level was found to be correlated with the GMT of this cortex. More specifically, a significant positive association was found between the T2-rest value of the LM at UL L3 (p = 0.004) and the GMT of the superior parietal cortex. Again, the factor group did not have any effect on these correlations (p > .05). Moreover, no association were recorded between the T2-rest of the LM at UL L4 and LL L4, and the GMT of the superior parietal cortex (p > .05).

As for the subcortical regions, the T2-rest of the LM at all lumbar levels was significantly positively associated with the GMT of the posterior cingulate cortex. More specifically, a significantly positive association was found between the T2-rest of LM at UL L3 (p = 0.005), UL L4 (p = 0.022) and LL L4 (p = 0.019), and the GMT of the posterior cingulate cortex after controlling for depression, health status and disability. However, patients with NSLBP did show significant higher GMT of the posterior cingulate cortex (p = 0.014) in this association with the T2-rest of the LM at UL L4 after controlling for depression, health status and disability. Nevertheless, the factor group did not effect the association between posterior cingulate cortex and the T2-rest of the LM at UL L3 and LL L4 (p > .05). Moreover, no association were detected between the T2-rest of the LM and the GMT of paracentral cortex (p > .05).

With respect to GMV of the frontal cortical regions, the T2-rest of the LM at the L4 levels was significantly positively correlated with GMV of the rostral middle frontal and caudal middle frontal cortices. More specifically, higher T2-rest values of the LM at LL L4 (p = 0.043) were related to higher GMV of the rostral middle frontal cortex. Furthermore, a significant positive association was found between the T2-rest of the LM at UL L4 (p = 0.041) and the GMV of the caudal middle frontal cortex after controlling for pain duration. However, the factor group had no effect on the correlation between the T2-rest of the LM and the GMV of the rostral middle frontal cortex or the caudal middle frontal cortex (p > .05). Moreover, no associations were found between the T2-rest of the LM muscle at other lumbar levels and the GMV of the rostral middle frontal and caudal middle frontal cortices (p > .05)

As for the middle cortical regions, the T2-rest of the LM at all lumbar levels was significantly positively associated with the GMV of the postcentral cortex. More specifically, a significantly positive association was found between the T2-rest of LM at UL L3 (p = 0.024), UL L4 (p = 0.011) and LL L4 (p = 0.001) and GMV of the postcentral cortex. The factor group did not affect these associations. Moreover, no significant correlations were found between the T2-rest and the GMV of the precentral and insular cortices (p > .05).

For the posterior cortical regions, the T2-rest of the LM at UL L4 (p = 0.023) was significantly positively associated with the GMV of the supramarginal cortex after controlling for pain catastrophizing and health status. However, the factor group showed no effect on these associations. Moreover, there was no significant correlation between the T2-rest of the LM muscle and GMV of the superior parietal cortex (p > .05).

For the subcortical region, the T2-rest of LM muscle was significantly associated with the GMV of the rostral anterior cingulate cortex. More specifically, there was a significantly positive association between the T2-rest of LM at UL L3 (p = 0.015), UL L4 (p = 0.007) and LL L4 (p = 0.001) and the GMV of the rostral anterior cingulate cortex after controlling for age and anxiety. However, the factor group showed no effect on these associations (p > .05).

### Association between the T2-shift of the LM muscle and GMT, GMV of the cortices

Results of the associations between the T2-shift of the LM and GMT and GMV of the sensorimotor cortices are presented in Table 6. A significantly negative association was found between the T2-shift at UL L4 and GMT of the supramarginal cortex (p = 0.048). However, the factor group showed no effect on this association (p > .05). Moreover, no associations were recorded between T2-shift of the LM and the GMT of other cortices (p > .05).

**Table 6:** Model characteristics and parameter estimates using linear mixed models for significant associations between T2-shift of LM, GMT and GMV of sensorimotor cortices.

	MODEL		PARAMETER ESTIMATES		SE	T	p-value
***T2-SHIFT***	***GMT***	**Rostral middle frontal (mm)**	Intercept	2.30	0.04	60.50	0.001
			Group	0.01	0.04	0.15	0.885
			UL L3 LM	0.00	0.00	1.00	0.322
			Intercept	2.33	0.04	55.97	0.001
			Group	0.01	0.04	0.15	0.881
			UL L4 LM	0.00	0.00	0.09	0.933
			Intercept	2.34	0.04	53.64	0.001
			Group	0.00	0.04	0.10	0.919
			LL L4 LM	0.01	0.00	−0.17	0.867
		**Caudal middle frontal (mm)**	Intercept	2.55	0.04	66.00	0.001
			Group	0.04	0.04	0.84	0.409
			UL L3 LM	0.00	0.00	0.00	0.997
			Intercept	2.58	0.04	63.86	0.001
			Group	0.03	0.04	0.64	0.527
			UL L4 LM	0.00	0.00	−1.07	0.293
			Intercept	2.59	0.04	61.32	0.001
			Group	0.02	0.04	0.59	0.560
			LL L4 LM	−0.01	0.00	−1.42	0.167
		**Precentral (mm)**	Intercept	2.64	0.04	66.06	0.001
			Group	0.01	0.05	0.12	0.905
			UL L3 LM	0.00	0.00	−0.10	0.923
			Intercept	2.63	0.04	61.52	0.001
			Group	0.01	0.05	0.20	0.843
			UL L4 LM	0.00	0.00	0.48	0.632
			Intercept	2.63	0.04	61.52	0.001
			Group	0.01	0.05	0.20	0.843
			LL L4 LM	0.00	0.00	0.48	0.632
		**Postcentral (mm)**	Intercept	2.13	0.04	61.13	0.001
			Group	−0.01	0.04	−0.21	0.837
			UL L3 LM	0.00	0.00	0.84	0.406
			Intercept	2.12	0.04	57.60	0.001
			Group	0.00	0.04	−0.04	0.969
			UL L4 LM	0.00	0.00	1.08	0.284
			Intercept	2.12	0.04	53.62	0.001
			Group	0.00	0.04	−0.09	0.929
			LL L4 LM	0.00	0.00	0.74	0.462
		**Insula (mm)**	Intercept	3.19	0.05	69.46	0.001
			Group	−0.04	0.05	−0.74	0.464
			UL L3 LM	0.00	0.00	−0.02	0.984
			Intercept	3.24	0.05	66.56	0.001
			Group	−0.05	0.05	−0.98	0.335
			UL L4 LM	−0.01	0.00	−1.62	0.112
			Intercept	3.23	0.05	63.12	0.001
			Group	−0.05	0.05	−0.93	0.362
			LL L4 LM	−0.01	0.00	−1.28	0.208
		**Superior parietal (mm)**	Intercept	2.22	0.04	62.63	0.001
			Group	−0.02	0.04	−0.53	0.602
			UL L3 LM	0.00	0.00	0.19	0.853
			Intercept	2.19	0.04	58.81	0.001
			Group	−0.01	0.04	−0.26	0.796
			UL L4 LM	0.00	0.00	1.88	0.066
			Intercept	2.20	0.04	56.13	0.001
			Group	−0.02	0.04	−0.35	0.730
			LL L4 LM	0.00	0.00	1.21	0.232
		**Supramarginal (mm)**	Intercept	2.63	0.04	63.97	0.001
			Group	0.05	0.05	1.06	0.300
			UL L3 LM	0.00	0.00	0.52	0.604
			Intercept	2.71	0.04	62.38	0.001
			Group	0.03	0.05	0.68	0.500
			***UL L4 LM***	−0.01	0.00	−2.03	0.048 *
			Intercept	2.69	0.04	61.38	0.001
			Group	0.04	0.04	0.86	0.400
			LL L4 LM	−0.01	0.00	−1.42	0.164
		**Paracentral (mm)**	Intercept	2.49	0.04	66.41	0.001
			Group	0.03	0.04	0.68	0.504
			UL L3 LM	0.00	0.00	−0.79	0.436
			Intercept	2.51	0.04	64.21	0.001
			Group	0.02	0.04	0.40	0.696
			UL L4 LM	0.00	0.00	−1.77	0.082
			Intercept	2.51	0.04	61.40	0.001
			Group	0.02	0.04	0.46	0.650
			LL L4 LM	0.01	0.00	−1.44	0.156
		**Posterior cingulate (mm)**	Intercept	2.66	0.04	64.90	0.001
			Group	−0.04	0.04	−0.90	0.376
			UL L3 LM	0.01	0.00	0.08	0.937
			Intercept	2.65	0.05	59.35	0.001
			Group	−0.04	0.05	−0.78	0.445
			UL L4 LM	0.01	0.00	0.51	0.617
			Intercept	2.66	0.05	57.44	0.001
			Group	−0.04	0.05	−0.85	0.406
			LL L4 LM	0.01	0.00	0.23	0.816
		**Rostral anterior cingulate (mm)**	Intercept	3.05	0.06	52.78	0.001
			Group	−0.04	0.06	−0.60	0.554
			UL L3 LM	0.01	0.01	−0.46	0.649
			Intercept	3.08	0.06	49.67	0.001
			Group	−0.05	0.06	−0.79	0.432
			UL L4 LM	−0.01	0.00	−1.04	0.303
			Intercept	3.03	0.07	46.24	0.001
			Group	−0.04	0.06	−0.58	0.566
			LL L4 LM	0.01	0.01	0.01	0.990
	***GMV***	***Rostral middle frontal (mm3)***	Intercept	15265.02	668.90	22.82	0.001
			Group	94.31	781.15	0.12	0.905
			UL L3 LM	61.84	46.41	1.33	0.189
			Intercept	15928.87	699.96	22.76	0.001
			Group	42.73	785.75	0.05	0.957
			UL L4 LM	−16.06	43.24	−0.37	0.712
			Intercept	16980.77	734.79	23.11	0.001
			Group	−204.77	803.46	−0.26	0.801
			***LL L4 LM***	−133.55	51.06	−2.57	0.013 *
		**Caudal middle frontal (mm3)**	Intercept	6136.49	440.49	13.93	0.001
			Group	827.91	493.75	1.68	0.106
			UL L3 LM	−2.47	33.05	−0.08	0.941
			Intercept	6013.68	467.03	12.88	0.001
			Group	856.82	501.67	1.71	0.100
			UL L4 LM	10.23	30.85	0.33	0.742
			Intercept	6159.37	490.10	12.57	0.001
			Group	817.83	498.35	1.64	0.114
			LL L4 LM	−4.73	38.06	−0.12	0.902
		**Precentral (mm3)**	Intercept	13283.10	509.77	26.06	0.001
			Group	254.12	595.52	0.43	0.673
			UL L3 LM	21.11	35.35	0.60	0.553
			Intercept	13082.83	534.32	24.49	0.001
			Group	355.53	607.84	0.59	0.564
			UL L4 LM	37.03	32.24	1.15	0.256
			Intercept	13303.49	563.46	23.61	0.001
			Group	288.32	604.85	0.48	0.638
			LL L4 LM	16.65	40.98	0.41	0.686
		**Postcentral (mm3)**	Intercept	9573.55	411.49	23.27	0.001
			Group	−14.14	459.87	−0.03	0.976
			UL L3 LM	13.99	31.02	0.45	0.654
			Intercept	9553.09	439.28	21.73	0.001
			Group	21.76	470.89	0.05	0.964
			UL L4 LM	13.36	29.10	0.46	0.648
			Intercept	10064.69	443.82	22.68	0.001
			Group	−106.64	446.57	−0.24	0.813
			LL L4 LM	−41.55	34.84	−1.19	0.240
		**Insula (mm3)**	Intercept	7005.54	267.56	26.18	0.001
			Group	−8.09	330.87	−0.02	0.981
			UL L3 LM	−17.17	15.97	−1.08	0.288
			Intercept	6973.66	274.51	25.40	0.001
			Group	−36.26	331.30	−0.11	0.914
			UL L4 LM	−10.72	14.54	−0.74	0.464
			Intercept	7004.72	286.56	24.45	0.001
			Group	−39.69	329.15	−0.12	0.905
			LL L4 LM	−15.27	18.68	−0.82	0.417
		**Superior parietal (mm3)**	Intercept	13243.86	399.93	33.12	0.001
			Group	−32.11	452.52	−0.07	0.944
			UL L3 LM	−34.04	29.52	−1.15	0.255
			Intercept	13237.43	423.19	31.28	0.001
			Group	−103.82	459.48	−0.23	0.823
			UL L4 LM	−26.91	27.54	−0.977	0.333
			Intercept	13328.11	439.98	30.29	0.001
			Group	−115.49	451.06	−0.26	0.800
			LL L4 LM	−39.74	33.86	−1.17	0.247
		**Supramarginal (mm3)**	Intercept	10727.42	434.82	24.67	0.001
			Group	271.60	473.86	0.57	0.572
			UL L3 LM	−16.42	34.09	−0.48	0.633
			Intercept	11075.94	450.03	24.61	0.001
			Group	138.99	464.34	0.30	0.767
			UL L4 LM	−47.99	31.26	−1.54	0.133
			Intercept	11115.64	460.32	24.15	0.001
			Group	147.58	448.93	0.33	0.745
			LL L4 LM	−57.43	37.24	−1.54	0.133
		***Paracentral (mm3)***	Intercept	3864.25	228.39	16.92	0.001
			Group	310.75	253.18	1.23	0.231
			UL L3 LM	−26.63	17.45	−1.53	0.135
			Intercept	3907.20	239.41	16.32	0.001
			Group	241.30	252.40	0.96	0.348
			UL L4 LM	−25.83	16.21	−1.59	0.118
			Intercept	4057.29	241.19	16.82	0.001
			Group	214.89	240.16	0.89	0.379
			***LL L4 LM***	−45.08	19.71	−2.36	0.024 *
		**Posterior cingulate (mm3)**	Intercept	3363.78	147.50	22.81	0.001
			Group	34.37	152.00	0.23	0.823
			UL L3 LM	−4.98	10.26	−0.49	0.630
			Intercept	3363.79	147.50	22.81	0.001
			Group	34.37	152.00	0.23	0.823
			UL L4 LM	−4.98	10.26	−0.47	0.630
			Intercept	3387.87	152.78	22.17	0.001
			Group	30.45	149.30	0.20	0.840
			LL L4 LM	−8.16	12.34	−0.66	0.513
		**Rostral anterior cingulate (mm3)**	Intercept	2503.97	145.47	17.21	0.001
			Group	98.16	155.02	0.63	0.529
			UL L3 LM	−1.18	11.76	−0.10	0.921
			Intercept	2502.11	157.33	15.90	0.001
			Group	96.13	158.18	0.61	0.546
			UL L4 LM	−0.77	11.24	−0.07	0.946
			Intercept	2556.69	164.37	15.55	0.001
			Group	83.28	157.46	0.51	0.599
			LL L4 LM	−6.86	13.51	−0.51	0.614

* p < .05, ** p < .01.

Legend: SE: standard error; t: t-value; CI: confidence interval, LM: lumbar multifidus, UL L3: upper-level of lumbar 3; UL L4: upper-level of lumbar 4; LL L4: lower-level of lumbar 4.

Furthermore, a significant negative association existed between the T2-shift of the LM muscle at LL L4 and the GMV of the rostral middle frontal (p = 0.013) and paracentral (p = 0.024) cortices. However, the factor group showed no effect on these associations (p > .05). No associations were recorded between the T2-shift of the LM and the GMV of other cortices (p > .05).

## Discussion

This study examined whether a relationship exists between structural and functional muscle characteristics of the LM and brain morphology of gray matter cortices related to sensorimotor function, and if having NSLBP influenced this relationship.

No relationship could be established between the macrostructural properties of the LM and the structural properties of the brain regions related to sensorimotor function, in neither NSLBP nor HC. However, this study does confirm relationships between structural properties of the brain regions related to sensorimotor function and microscopic properties of the LM related to fat infiltrations and fiber typing. Firstly, a larger volume of the paracentral and the postcentral cortex is related to deterioration the muscle structural quality of the LM, irrespective of the presence of LBP. Secondly, a higher thickness and larger volume of cortices related to sensorimotor function is associated with higher proportions of oxidative slow twitch fibers in the LM muscle, irrespective of the presence of LBP. This can be observed by the increased GMT of the rostral middle frontal, caudal middle frontal, precentral, postcentral and posterior cingulate cortices which are related to a higher T2-rest of the LM. Moreover, when focusing on the lean LM muscle, a higher T2-rest value is also associated with a larger volume of the precentral, postcentral and posterior cingulate cortices. Despite the positive relationships between the thickness and volume of these cortices and the T2-rest of LM, patients with NSLBP showed an increased thickness of the caudal middle frontal and posterior cingulate cortices, which in turn can be associated with fiber typing related to slow twitch muscle fibers in the LM. Lastly, with respect to muscle functional properties of the LM, when a higher metabolic activity of LM was observed this was associated with increased GMT of the supramarginal cortex and larger GMV of the rostral middle frontal and paracentral cortices. However, this relationship was not mediated by the presence of NSLBP.

It can be assumed that besides muscle structure, physiological and psychosocial factors can also influence the structural properties of the brain regions related to sensorimotor function. This was confirmed in the current study that current LBP intensity, pain duration, pain catastrophizing, anxiety and health status were of influence factors on the changes in the regional thickness and volume of the cortices than muscle CSA and fat CSA of the LM muscle. Furthermore, metabolic and hormonal factors, including hormonal status, vitamin D, and calcium levels, may also influence these brain-spine associations [81]. These biochemical parameters can affect both cortical structure and spinal health, suggesting that the observed relationships may be modulated by multiple interacting physiological systems beyond the musculoskeletal components examined in this study.

This study revealed that a higher MFI of the LM is related to a high volume of the paracentral and postcentral cortices, in healthy people as well as those with NSLBP. The MFI reflects the amount of fatty infiltrations within the lean muscle tissue, which affects the muscle its function and performance. Furthermore, it is known that the primary somatosensory cortex is located in the postcentral cortex and responsible for proprioceptive processing such as somatic sensations for the lower limbs or torso [82–84]. As supplementary motor area is part of the paracentral cortex known and involved in programming (rehearsal) complex sequences and coordinating of bilateral body movements [85]. In addition, the paracentral cortex is involved in the transformation of kinematic to dynamic information. For instance, dynamic movement plans are represented in kinematic terms, in order to instruct appropriate muscles to contract with appropriate force [86]. Considering that both paracentral and postcentral cortex as important regions for sensorimotor function, it might not be surprising that alteration of LM function due to the poor quality of the lean muscle tissue are associated with increased volumes of these cortices. However, this is the first study to establish a relationship between the MFI and the volume of the paracentral and postcentral cortices, thus further research is warranted to confirm this assumption.

The current study reveals a positive association between the T2-rest of the LM muscle at multiple levels of the lumbar spine and GMT of the rostral middle frontal, caudal middle frontal, precentral, superior parietal, and posterior cingulate cortices. This finding could offer a new insight into the relationship between alterations of fiber type characteristics and thickness of brain region’s related to sensorimotor function. A higher T2-rest reflects a higher proportion of oxidative fiber type [23,24]. Apart from that, the LM muscle is a tonic muscle that normally performs endurance and aerobic contractions of the muscle [62,87]. Therefore, obtaining higher amounts of oxidative slow twitch fibers within muscle is desirable to its function, and could be one of the factors to consider when one would aim reversing the decreased thickness of these cortices.

Furthermore, it is interesting to discover that the relationship between the T2-rest and the GMT of the caudal middle frontal and posterior cingulate cortex is mediated by the presence of LBP. Both cortices are involved in pain and cognitive processes. Previous studies have shown that the caudal middle frontal cortex which is situated in the dorsal lateral prefrontal cortex, show a significant role in controlling pain perception [88] by modulating the cortical and subcortical pathways [89]. Moreover, other studies have shown that a decrease in the thickness of the caudal middle frontal cortex can be seen, for example as in populations with ongoing pain [90]. The posterior cingulate cortex has a dense structural connectivity with the major node of the brain regions, involved in cognitive activities and pain attention [91]. Moreover, it is involved in the posterior subnetwork of the default mode network, which implicates consciousness and memory processing [92,93]. A fiber typing determined by the T2-rest value is one of a method to determine structural microscopic properties of the of the LM. Lower T2-rest values in the LM reflecting a higher proportion of glycolytic fibers have been shown in LBP patients compared to healthy people [23]. Changes in thickness of the caudal middle frontal and the posterior cingulate cortices may be mediated by changes in the fiber type composition of LM. Therefore, treatment directed to improve muscle fiber transformation into oxidative fibers by means of strength and endurance training may be adequate to increase the proportion and size of slow-twitch muscle fibers may be effective to increase the thickness of both cortices in NSLBP patients.

This study found a negative association between the T2-shift of the LM at L4 and the thickness of the supramarginal cortex and the volume of the rostral middle frontal and paracentral cortex no group effects were established. The results suggest that a higher metabolic activity of the LM is related to a smaller volume of these cortices. The T2-shift value of LM represents the amount of the performed muscle activity during contraction. A higher T2-shift during a standardized activity resembles a higher metabolic change in the muscle, hence resulting in more rapid muscle fatigue. Therefore, the T2-shift of the LM muscle is an indication of the efficiency of muscle recruitment contraction [23]. Hence, the results show that the degree to which the LM works efficiently is related to the thickness of supramarginal cortex and the rostral middle frontal and paracentral cortex volume.

These study findings have important clinical implications for understanding and managing NSLBP. The demonstrated associations between lumbar multifidus morphology and function with cortical gray matter structure suggest that NSLBP involves complex brain-muscle interactions beyond localized spinal pathology. Clinically, this supports the rationale for multimodal treatment approaches that address both peripheral muscle dysfunction and central nervous system changes. The positive associations between muscle fat infiltration (MFI) and cortical volume in sensorimotor regions, along with the relationships between muscle metabolic activity (T2-rest and T2-shift) and cortical thickness, indicate that interventions targeting muscle quality and function may influence cortical reorganization. This knowledge could guide the development of rehabilitation strategies that combine targeted lumbar muscle training with approaches addressing cortical plasticity, such as motor imagery, sensorimotor retraining, or neurofeedback. Furthermore, these findings suggest that muscle MRI parameters could potentially serve as biomarkers for predicting treatment response or monitoring neuroplastic changes during rehabilitation. The observation that some associations occur irrespective of LBP status highlights the importance of preventing muscle degeneration before pain develops, emphasizing early intervention strategies in at-risk populations.

A limitation of this study is that the NSLBP patients had low self-reported pain intensities as well as pain duration variance related to LBP and low presence of psychological factors such as pain catastrophizing, anxiety and depression. This shows that most of the participants in this study did not experience intense pain sensations, variable duration of LBP symptoms (range of 3 months to 204 months) or less influence by psychological problems. Additionally, the time limit criteria for LBP inclusion may have biased the results, as modifications of the gray matter cortex require substantial time to adapt to current conditions. Moreover, the group effect not being significant in most of the relationships between the muscle structure and function and brain outcomes might have been caused by the small sample size. A priori power calculation revealed that a minimum 24 subjects were needed. For both NSLBP and HC groups, this amount unfortunately was not reached. Therefore, a replication of similar correlations between the muscle and brain with a larger sample size is warranted in future study. The focus on multifidus muscle alone may be a conceptual limitation, as recent evidence highlights the potentially greater role of erector spinae in LBP pathogenesis [94,95]. Future studies should assess both muscle groups to comprehensively evaluate paraspinal contributions to NSLBP. The absence of central sensitization screening tools (such as the Central Sensitization Inventory or quantitative sensory testing) means we cannot determine whether our findings are confounded by this important pain mechanism. Future studies should incorporate central sensitization assessments to better characterize the study population and interpret brain-muscle relationships. Absence of standardized physical activity assessment limits our ability to control for activity-related confounding on muscle morphology, potentially introducing unmeasured variability. Future studies should employ validated activity assessment tools.

Finally, the present study is of a correlative nature. The significant correlations between the muscle structure, function and the brain morphology provided us information on the general associations between these variables but did not allow us to test aspect of strong causality. To achieve this, it is recommended that a prospective and longitudinal study be carried out in the future.

## Conclusion

In conclusion, this study did find certain relationships between muscle structure or function of the LM and brain structural characteristics in both NSLBP and HC. Regarding muscles structure, low muscle quality of the LM tissue (which is expressed by a high MFI) was associated with a larger volume of the postcentral and paracentral cortices. Noteworthy, the latter observation was not mediated by the presence of LBP. No associations were found between the CSA of the fatty tissue or the lean muscle CSA and the brain structural properties. With respect to resting metabolic tissue characteristics of the LM, higher T2-rest values of the LM at different lumbar levels were associated with higher thickness of the rostral middle frontal, caudal middle frontal, precentral, superior pariental, and posterior cingulate cortices, and a larger volume of the rostral middle frontal, caudal middle frontal, postcentral, supramarginal and rostral anterior cingulate cortices, indicating that a higher resting metabolic state of the LM (with higher rates begin indicative of higher proportions of type I than type II fibres) is related to higher GMT at the brains sensorimotor regions. Only the relationship between a higher T2-rest of the LM with a thicker caudal middle frontal and larger posterior cingulate cortex volume was mediated by the presence of NSLBP. When muscle function was assessed based on the metabolic activity of the LM, both NSLBP patients as well HC, showed that the degree to which the muscle works efficiently is inversely related to a thinner supramarginal cortex and smaller volumes of the rostral middle frontal and paracentral cortices. These innovative findings suggest that relationships between brain structural properties can underly changes in muscle structural/functional properties of the LM muscle, the latter being observed in NSLBP by several studies. The current study showed that the presence of NSLBP can indeed mediate the relationship between LM muscle structure/function and GMT/GMV of brain regions involved in sensorimotor processing and control. Longitudinal research including larger samples of NSLBP patients are warranted to further unravel the impact of LBP on the relationship between LM muscle structural and functional characteristics and brain morphology.

## References

[1] Becker A, Held H, Redaelli M, Strauch K, Chenot JF, Leonhardt C, et al. Low back pain in primary care: costs of care and prediction of future health care utilization. Spine (Phila Pa 1976). 2010;35(18):1714–20. doi: 10.1097/brs.0b013e3181cd656f 21374895

[2] Hestbaek L, Leboeuf-Yde C, Manniche C. Low back pain: what is the long-term course? A review of studies of general patient populations. Eur Spine J. 2003;12(2):149–65. doi: 10.1007/s00586-002-0508-5 12709853 PMC3784852

[3] Ferreira ML, et al. Global, regional, and national burden of low back pain, 1990–2020, its attributable risk factors, and projections to 2050: a systematic analysis of the Global Burden of Disease Study 2021. The Lancet Rheumatology. 2023;5(6):e316–29. 10.1016/S2665-9913(23)00098-X PMC10234592 37273833

[4] Von Korff M. Studying the natural history of back pain. Spine (Phila Pa 1976). 1994;19(18 Suppl):2041S-2046S. doi: 10.1097/00007632-199409151-00005 7801181

[5] Airaksinen O, Brox JI, Cedraschi C, Hildebrandt J, Klaber-Moffett J, Kovacs F, et al. Chapter 4. European guidelines for the management of chronic nonspecific low back pain. Eur Spine J. 2006;15 Suppl 2(Suppl 2):S192-300. doi: 10.1007/s00586-006-1072-1 16550448 PMC3454542

[6] Hallegraeff JM, Krijnen WP, van der Schans CP, de Greef MHG. Expectations about recovery from acute non-specific low back pain predict absence from usual work due to chronic low back pain: a systematic review. J Physiother. 2012;58(3):165–72. doi: 10.1016/S1836-9553(12)70107-8 22884183

[7] Apkarian AV, Bushnell MC, Treede R-D, Zubieta J-K. Human brain mechanisms of pain perception and regulation in health and disease. Eur J Pain. 2005;9(4):463–84. doi: 10.1016/j.ejpain.2004.11.001 15979027

[8] Schmidt-Wilcke T, Leinisch E, Gänssbauer S, Draganski B, Bogdahn U, Altmeppen J, et al. Affective components and intensity of pain correlate with structural differences in gray matter in chronic back pain patients. Pain. 2006;125(1–2):89–97. doi: 10.1016/j.pain.2006.05.004 16750298

[9] Etkin A, Egner T, Kalisch R. Emotional processing in anterior cingulate and medial prefrontal cortex. Trends Cogn Sci. 2011;15(2):85–93. doi: 10.1016/j.tics.2010.11.004 21167765 PMC3035157

[10] Brumagne S, Diers M, Danneels L, Moseley GL, Hodges PW. Neuroplasticity of Sensorimotor Control in Low Back Pain. J Orthop Sports Phys Ther. 2019;49(6):402–14. doi: 10.2519/jospt.2019.8489 31151373

[11] Rocca MA, Ceccarelli A, Falini A, Colombo B, Tortorella P, Bernasconi L, et al. Brain gray matter changes in migraine patients with T2-visible lesions: a 3-T MRI study. Stroke. 2006;37(7):1765–70. doi: 10.1161/01.STR.0000226589.00599.4d 16728687

[12] Apkarian AV, Sosa Y, Sonty S, Levy RM, Harden RN, Parrish TB, et al. Chronic back pain is associated with decreased prefrontal and thalamic gray matter density. J Neurosci. 2004;24(46):10410–5. doi: 10.1523/JNEUROSCI.2541-04.2004 15548656 PMC6730296

[13] Kim JH, Suh S-I, Seol HY, Oh K, Seo W-K, Yu S-W, et al. Regional grey matter changes in patients with migraine: a voxel-based morphometry study. Cephalalgia. 2008;28(6):598–604. doi: 10.1111/j.1468-2982.2008.01550.x 18422725

[14] Kuchinad A, Schweinhardt P, Seminowicz DA, Wood PB, Chizh BA, Bushnell MC. Accelerated brain gray matter loss in fibromyalgia patients: premature aging of the brain?. J Neurosci. 2007;27(15):4004–7. doi: 10.1523/JNEUROSCI.0098-07.2007 17428976 PMC6672521

[15] Mao C, Wei L, Zhang Q, Liao X, Yang X, Zhang M. Differences in brain structure in patients with distinct sites of chronic pain: A voxel-based morphometric analysis. Neural Regen Res. 2013;8(32):2981–90. doi: 10.3969/j.issn.1673-5374.2013.32.001 25206618 PMC4146206

[16] Ivo R, Nicklas A, Dargel J, Sobottke R, Delank K-S, Eysel P, et al. Brain structural and psychometric alterations in chronic low back pain. Eur Spine J. 2013;22(9):1958–64. doi: 10.1007/s00586-013-2692-x 23392554 PMC3777081

[17] Caeyenberghs K, Pijnenburg M, Goossens N, Janssens L, Brumagne S. Associations between Measures of Structural Morphometry and Sensorimotor Performance in Individuals with Nonspecific Low Back Pain. AJNR Am J Neuroradiol. 2017;38(1):183–91. doi: 10.3174/ajnr.A5020 27884877 PMC7963671

[18] Seminowicz DA, Wideman TH, Naso L, Hatami-Khoroushahi Z, Fallatah S, Ware MA, et al. Effective treatment of chronic low back pain in humans reverses abnormal brain anatomy and function. J Neurosci. 2011;31(20):7540–50. doi: 10.1523/JNEUROSCI.5280-10.2011 21593339 PMC6622603

[19] Brumagne S, Janssens L, Knapen S, Claeys K, Suuden-Johanson E. Persons with recurrent low back pain exhibit a rigid postural control strategy. Eur Spine J. 2008;17(9):1177–84. doi: 10.1007/s00586-008-0709-7 18594876 PMC2527415

[20] Steffens D, Maher CG, Pereira LSM, Stevens ML, Oliveira VC, Chapple M, et al. Prevention of Low Back Pain: A Systematic Review and Meta-analysis. JAMA Intern Med. 2016;176(2):199–208. doi: 10.1001/jamainternmed.2015.7431 26752509

[21] Hodges PW, Tucker K. Moving differently in pain: a new theory to explain the adaptation to pain. Pain. 2011;152(3 Suppl):S90–8. doi: 10.1016/j.pain.2010.10.020 21087823

[22] Hodges PW, Danneels L. Changes in Structure and Function of the Back Muscles in Low Back Pain: Different Time Points, Observations, and Mechanisms. J Orthop Sports Phys Ther. 2019;49(6):464–76. doi: 10.2519/jospt.2019.8827 31151377

[23] D’hooge R, Cagnie B, Crombez G, Vanderstraeten G, Achten E, Danneels L. Lumbar muscle dysfunction during remission of unilateral recurrent nonspecific low-back pain: evaluation with muscle functional MRI. Clin J Pain. 2013;29(3):187–94. doi: 10.1097/AJP.0b013e31824ed170 23369927

[24] Goubert D, De Pauw R, Meeus M, Willems T, Cagnie B, Schouppe S, et al. Lumbar muscle structure and function in chronic versus recurrent low back pain: a cross-sectional study. Spine J. 2017;17(9):1285–96. doi: 10.1016/j.spinee.2017.04.025 28456669

[25] Hodges PW, Moseley GL. Pain and motor control of the lumbopelvic region: effect and possible mechanisms. J Electromyogr Kinesiol. 2003;13(4):361–70. doi: 10.1016/s1050-6411(03)00042-7 12832166

[26] MacDonald D, Moseley LG, Hodges PW. Why do some patients keep hurting their back? Evidence of ongoing back muscle dysfunction during remission from recurrent back pain. Pain. 2009;142(3):183–8. doi: 10.1016/j.pain.2008.12.002 19186001

[27] D’hooge R, Cagnie B, Crombez G, Vanderstraeten G, Dolphens M, Danneels L. Increased intramuscular fatty infiltration without differences in lumbar muscle cross-sectional area during remission of unilateral recurrent low back pain. Man Ther. 2012;17(6):584–8. doi: 10.1016/j.math.2012.06.007 22784801

[28] Danneels LA, Vanderstraeten GG, Cambier DC, Witvrouw EE, De Cuyper HJ. CT imaging of trunk muscles in chronic low back pain patients and healthy control subjects. Eur Spine J. 2000;9(4):266–72. doi: 10.1007/s005860000190 11261613 PMC3611341

[29] Goubert D, Oosterwijck JV, Meeus M, Danneels L. Structural Changes of Lumbar Muscles in Non-specific Low Back Pain: A Systematic Review. Pain Physician. 2016;19(7):E985–1000. doi: 10.36076/ppj/2016.19.e985 27676689

[30] Hodges PW, Galea MP, Holm S, Holm AK. Corticomotor excitability of back muscles is affected by intervertebral disc lesion in pigs. Eur J Neurosci. 2009;29(7):1490–500. doi: 10.1111/j.1460-9568.2009.06670.x 19519631

[31] Tsao H, Danneels LA, Hodges PW. ISSLS prize winner: Smudging the motor brain in young adults with recurrent low back pain. Spine (Phila Pa 1976). 2011;36(21):1721–7. doi: 10.1097/BRS.0b013e31821c4267 21508892

[32] Alnojeidi AH. Gender differences in low back pain and self-reported muscle strengthening activity among US adults. 2015.

[33] Gupta A, Mayer EA, Fling C, Labus JS, Naliboff BD, Hong J-Y, et al. Sex-based differences in brain alterations across chronic pain conditions. J Neurosci Res. 2017;95(1–2):604–16. doi: 10.1002/jnr.23856 27870423 PMC5120652

[34] Balagué F, Mannion AF, Pellisé F, Cedraschi C. Non-specific low back pain. Lancet. 2012;379(9814):482–91. doi: 10.1016/S0140-6736(11)60610-7 21982256

[35] Jensen MP, Turner JA, Romano JM. What is the maximum number of levels needed in pain intensity measurement? Pain. 1994;58(3):387–92. doi: 10.1016/0304-3959(94)90133-3 7838588

[36] Spinhoven P, Ormel J, Sloekers PP, Kempen GI, Speckens AE, Van Hemert AM. A validation study of the Hospital Anxiety and Depression Scale (HADS) in different groups of Dutch subjects. Psychol Med. 1997;27(2):363–70. doi: 10.1017/s0033291796004382 9089829

[37] Bjelland I, Dahl AA, Haug TT, Neckelmann D. The validity of the Hospital Anxiety and Depression Scale. An updated literature review. J Psychosom Res. 2002;52(2):69–77. doi: 10.1016/s0022-3999(01)00296-3 11832252

[38] Sullivan MJL, Bishop SR, Pivik J. The Pain Catastrophizing Scale: Development and validation. Psychological Assessment. 1995;7(4):524–32. doi: 10.1037/1040-3590.7.4.524

[39] Van Damme S, et al. De pain catastrophizing scale: psychometrische karakteristieken en normering. Gedragstherapie. 2000;33(3):209–20.

[40] Fischl B. FreeSurfer. Neuroimage. 2012;62(2):774–81. 22248573 10.1016/j.neuroimage.2012.01.021 PMC3685476

[41] Fischl B, Salat DH, Busa E, Albert M, Dieterich M, Haselgrove C, et al. Whole brain segmentation: automated labeling of neuroanatomical structures in the human brain. Neuron. 2002;33(3):341–55. doi: 10.1016/s0896-6273(02)00569-x 11832223

[42] Ségonne F, Dale AM, Busa E, Glessner M, Salat D, Hahn HK, et al. A hybrid approach to the skull stripping problem in MRI. Neuroimage. 2004;22(3):1060–75. doi: 10.1016/j.neuroimage.2004.03.032 15219578

[43] Fischl B, Salat DH, van der Kouwe AJW, Makris N, Ségonne F, Quinn BT, et al. Sequence-independent segmentation of magnetic resonance images. Neuroimage. 2004;23 Suppl 1:S69-84. doi: 10.1016/j.neuroimage.2004.07.016 15501102

[44] Sled JG, Zijdenbos AP, Evans AC. A nonparametric method for automatic correction of intensity nonuniformity in MRI data. IEEE Transactions on Medical Imaging. 1998;17(1):87–97. 9617910 10.1109/42.668698

[45] Fischl B, Liu A, Dale AM. Automated manifold surgery: constructing geometrically accurate and topologically correct models of the human cerebral cortex. IEEE Transactions on Medical Imaging. 2001;20(1):70–80. 11293693 10.1109/42.906426

[46] Ségonne F, Pacheco J, Fischl B. Geometrically accurate topology-correction of cortical surfaces using nonseparating loops. IEEE Trans Med Imaging. 2007;26(4):518–29. doi: 10.1109/TMI.2006.887364 17427739

[47] Dale AM, Fischl B, Sereno MI. Cortical surface-based analysis. I. Segmentation and surface reconstruction. Neuroimage. 1999;9(2):179–94. doi: 10.1006/nimg.1998.0395 9931268

[48] Dale AM, Sereno MI. Improved Localizadon of Cortical Activity by Combining EEG and MEG with MRI Cortical Surface Reconstruction: A Linear Approach. J Cogn Neurosci. 1993;5(2):162–76. doi: 10.1162/jocn.1993.5.2.162 23972151

[49] Fischl B, Dale AM. Measuring the thickness of the human cerebral cortex from magnetic resonance images. Proc Natl Acad Sci U S A. 2000;97(20):11050–5. doi: 10.1073/pnas.200033797 10984517 PMC27146

[50] Desikan RS, Ségonne F, Fischl B, Quinn BT, Dickerson BC, Blacker D, et al. An automated labeling system for subdividing the human cerebral cortex on MRI scans into gyral based regions of interest. Neuroimage. 2006;31(3):968–80. doi: 10.1016/j.neuroimage.2006.01.021 16530430

[51] Yang Q, Wang Z, Yang L, Xu Y, Chen LM. Cortical thickness and functional connectivity abnormality in chronic headache and low back pain patients. Hum Brain Mapp. 2017;38(4):1815–32. doi: 10.1002/hbm.23484 28052444 PMC6867133

[52] Meier ML, Vrana A, Schweinhardt P. Low Back Pain: The Potential Contribution of Supraspinal Motor Control and Proprioception. Neuroscientist. 2019;25(6):583–96. doi: 10.1177/1073858418809074 30387689 PMC6900582

[53] Lhomond O, Teasdale N, Simoneau M, Mouchnino L. Supplementary Motor Area and Superior Parietal Lobule Restore Sensory Facilitation Prior to Stepping When a Decrease of Afferent Inputs Occurs. Front Neurol. 2019;9:1132. doi: 10.3389/fneur.2018.01132 30662426 PMC6328453

[54] Vrana A. Differential neural processing during motor imagery of daily activities in chronic low back pain patients. PLoS One. 2015;10(11):e0142391. 10.1371/journal.pone.0142391 PMC4646462 26569602

[55] Čeko M, Shir Y, Ouellet JA, Ware MA, Stone LS, Seminowicz DA. Partial recovery of abnormal insula and dorsolateral prefrontal connectivity to cognitive networks in chronic low back pain after treatment. Hum Brain Mapp. 2015;36(6):2075–92. doi: 10.1002/hbm.22757 25648842 PMC6869701

[56] Silfies SP, Beattie P, Jordon M, Vendemia JMC. Assessing sensorimotor control of the lumbopelvic-hip region using task-based functional MRI. J Neurophysiol. 2020;124(1):192–206. doi: 10.1152/jn.00288.2019 32519579

[57] Morrison I, Peelen MV, Downing PE. The sight of others’ pain modulates motor processing in human cingulate cortex. Cerebral Cortex. 2006;17(9):2214–22. 17124286 10.1093/cercor/bhl129

[58] Elliott JM, Pedler AR, Jull GA, Van Wyk L, Galloway GG, OʼLeary SP. Differential changes in muscle composition exist in traumatic and nontraumatic neck pain. Spine (Phila Pa 1976). 2014;39(1):39–47. doi: 10.1097/BRS.0000000000000033 24270932

[59] Wokke BH, Bos C, Reijnierse M, van Rijswijk CS, Eggers H, Webb A, et al. Comparison of dixon and T1-weighted MR methods to assess the degree of fat infiltration in duchenne muscular dystrophy patients. J Magn Reson Imaging. 2013;38(3):619–24. doi: 10.1002/jmri.23998 23292884

[60] Dickx N, Cagnie B, Achten E, Vandemaele P, Parlevliet T, Danneels L. Changes in lumbar muscle activity because of induced muscle pain evaluated by muscle functional magnetic resonance imaging. Spine (Phila Pa 1976). 2008;33(26):E983-9. doi: 10.1097/BRS.0b013e31818917d0 19092609

[61] Dickx N, D’Hooge R, Cagnie B, Deschepper E, Verstraete K, Danneels L. Magnetic resonance imaging and electromyography to measure lumbar back muscle activity. Spine (Phila Pa 1976). 2010;35(17):E836-42. doi: 10.1097/BRS.0b013e3181d79f02 20628333

[62] Dickx N, Cagnie B, Achten E, Vandemaele P, Parlevliet T, Danneels L. Differentiation between deep and superficial fibers of the lumbar multifidus by magnetic resonance imaging. Eur Spine J. 2010;19(1):122–8. doi: 10.1007/s00586-009-1171-x 19777271 PMC2899729

[63] Cagnie B, Elliott JM, O’Leary S, D’hooge R, Dickx N, Danneels LA. Muscle functional MRI as an imaging tool to evaluate muscle activity. J Orthop Sports Phys Ther. 2011;41(11):896–903. doi: 10.2519/jospt.2011.3586 21891877

[64] Meyer RA, Prior BM. Functional magnetic resonance imaging of muscle. Exerc Sport Sci Rev. 2000;28(2):89–92. 10902092

[65] Mayer JM, Graves JE, Clark BC, Formikell M, Ploutz-Snyder LL. The use of magnetic resonance imaging to evaluate lumbar muscle activity during trunk extension exercise at varying intensities. Spine (Phila Pa 1976). 2005;30(22):2556–63. doi: 10.1097/01.brs.0000186321.24370.4b 16284595

[66] Danneels LA, Vanderstraeten GG, Cambier DC, Witvrouw EE, Bourgois J, Dankaerts W, et al. Effects of three different training modalities on the cross sectional area of the lumbar multifidus muscle in patients with chronic low back pain. Br J Sports Med. 2001;35(3):186–91. doi: 10.1136/bjsm.35.3.186 11375879 PMC1724339

[67] De Ridder EMD, Van Oosterwijck JO, Vleeming A, Vanderstraeten GG, Danneels LA. Muscle functional MRI analysis of trunk muscle recruitment during extension exercises in asymptomatic individuals. Scand J Med Sci Sports. 2015;25(2):196–204. doi: 10.1111/sms.12190 24605781

[68] Kalichman L, Hodges P, Li L, Guermazi A, Hunter DJ. Changes in paraspinal muscles and their association with low back pain and spinal degeneration: CT study. Eur Spine J. 2010;19(7):1136–44. doi: 10.1007/s00586-009-1257-5 20033739 PMC2900015

[69] Cooper RG, St Clair Forbes W, Jayson MI. Radiographic demonstration of paraspinal muscle wasting in patients with chronic low back pain. Br J Rheumatol. 1992;31(6):389–94. doi: 10.1093/rheumatology/31.6.389 1534505

[70] Crossman K, Mahon M, Watson PJ, Oldham JA, Cooper RG. Chronic low back pain-associated paraspinal muscle dysfunction is not the result of a constitutionally determined “adverse” fiber-type composition. Spine (Phila Pa 1976). 2004;29(6):628–34. doi: 10.1097/01.brs.0000115133.97216.ec 15014272

[71] Teichtahl AJ, Urquhart DM, Wang Y, Wluka AE, O’Sullivan R, Jones G, et al. Physical inactivity is associated with narrower lumbar intervertebral discs, high fat content of paraspinal muscles and low back pain and disability. Arthritis Res Ther. 2015;17(1):114. doi: 10.1186/s13075-015-0629-y 25947906 PMC4422596

[72] Kamaz M, Kireşi D, Oğuz H, Emlik D, Levendoğlu F. CT measurement of trunk muscle areas in patients with chronic low back pain. Diagn Interv Radiol. 2007;13(3):144–8. 17846989

[73] Fortin M, Videman T, Gibbons LE, Battié MC. Paraspinal muscle morphology and composition: a 15-yr longitudinal magnetic resonance imaging study. Med Sci Sports Exerc. 2014;46(5):893–901. doi: 10.1249/MSS.0000000000000179 24091994

[74] Geisser ME, Haig AJ, Wallbom AS, Wiggert EA. Pain-related fear, lumbar flexion, and dynamic EMG among persons with chronic musculoskeletal low back pain. Clin J Pain. 2004;20(2):61–9. doi: 10.1097/00002508-200403000-00001 14770044

[75] Shigetoh H, Nishi Y, Osumi M, Morioka S. Combined abnormal muscle activity and pain-related factors affect disability in patients with chronic low back pain: An association rule analysis. PLoS One. 2020;15(12):e0244111. doi: 10.1371/journal.pone.0244111 33332431 PMC7746291

[76] Bingel U, Tracey I. Imaging CNS modulation of pain in humans. Physiology (Bethesda). 2008;23:371–80. doi: 10.1152/physiol.00024.2008 19074744

[77] Davis KD, Moayedi M. Central mechanisms of pain revealed through functional and structural MRI. J Neuroimmune Pharmacol. 2013;8(3):518–34. doi: 10.1007/s11481-012-9386-8 22825710

[78] Seminowicz DA, Shpaner M, Keaser ML, Krauthamer GM, Mantegna J, Dumas JA, et al. Cognitive-behavioral therapy increases prefrontal cortex gray matter in patients with chronic pain. J Pain. 2013;14(12):1573–84. doi: 10.1016/j.jpain.2013.07.020 24135432 PMC3874446

[79] Freund P, Weiskopf N, Ward NS, Hutton C, Gall A, Ciccarelli O, et al. Disability, atrophy and cortical reorganization following spinal cord injury. Brain. 2011;134(Pt 6):1610–22. doi: 10.1093/brain/awr093 21586596 PMC3102242

[80] Chowdhury MZI, Turin TC. Variable selection strategies and its importance in clinical prediction modelling. Fam Med Community Health. 2020;8(1):e000262. doi: 10.1136/fmch-2019-000262 32148735 PMC7032893

[81] Ekşi MŞ, Orhun Ö, Demir YN, Kara M, Berikol G, Özcan-Ekşi EE. Are serum thyroid hormone, parathormone, calcium, and vitamin D levels associated with lumbar spine degeneration? A cross-sectional observational clinical study. Eur Spine J. 2023;32(5):1561–74. doi: 10.1007/s00586-023-07673-w 36976340

[82] Kropf E, Syan SK, Minuzzi L, Frey BN. From anatomy to function: the role of the somatosensory cortex in emotional regulation. Braz J Psychiatry. 2019;41(3):261–9. doi: 10.1590/1516-4446-2018-0183 30540029 PMC6794131

[83] Lloyd DM, McGlone FP, Yosipovitch G. Somatosensory pleasure circuit: from skin to brain and back. Exp Dermatol. 2015;24(5):321–4. doi: 10.1111/exd.12639 25607755

[84] Roux F-E, Djidjeli I, Durand J-B. Functional architecture of the somatosensory homunculus detected by electrostimulation. J Physiol. 2018;596(5):941–56. doi: 10.1113/JP275243 29285773 PMC5830421

[85] Gerloff C, Corwell B, Chen R, Hallett M, Cohen LG. Stimulation over the human supplementary motor area interferes with the organization of future elements in complex motor sequences. Brain. 1997;120 ( Pt 9):1587–602. doi: 10.1093/brain/120.9.1587 9313642

[86] Sergio LE, Hamel-Pâquet C, Kalaska JF. Motor cortex neural correlates of output kinematics and kinetics during isometric-force and arm-reaching tasks. Journal of Neurophysiology. 2005;94(4):2353–78. 15888522 10.1152/jn.00989.2004

[87] Bonny JM, Zanca M, Boespflug-Tanguy O, Dedieu V, Joandel S, Renou JP. Characterization in vivo of muscle fiber types by magnetic resonance imaging. Magn Reson Imaging. 1998;16(2):167–73. doi: 10.1016/s0730-725x(97)00249-x 9508273

[88] Petrovic P, Ingvar M. Imaging cognitive modulation of pain processing. Pain. 2002;95(1–2):1–5. doi: 10.1016/s0304-3959(01)00467-5 11790461

[89] Lorenz J, Minoshima S, Casey KL. Keeping pain out of mind: the role of the dorsolateral prefrontal cortex in pain modulation. Brain. 2003;126(Pt 5):1079–91. doi: 10.1093/brain/awg102 12690048

[90] Ruscheweyh R, Deppe M, Lohmann H, Stehling C, Flöel A, Ringelstein BE, et al. Pain is associated with regional grey matter reduction in the general population. Pain. 2011;152(4):904–11. doi: 10.1016/j.pain.2011.01.013 21296501

[91] Leech R, Braga R, Sharp DJ. Echoes of the brain within the posterior cingulate cortex. J Neurosci. 2012;32(1):215–22. doi: 10.1523/JNEUROSCI.3689-11.2012 22219283 PMC6621313

[92] Frøkjær JB, Bouwense SAW, Olesen SS, Lundager FH, Eskildsen SF, van Goor H, et al. Reduced cortical thickness of brain areas involved in pain processing in patients with chronic pancreatitis. Clin Gastroenterol Hepatol. 2012;10(4):434-8.e1. doi: 10.1016/j.cgh.2011.11.024 22155560

[93] Hemington KS, Wu Q, Kucyi A, Inman RD, Davis KD. Abnormal cross-network functional connectivity in chronic pain and its association with clinical symptoms. Brain Struct Funct. 2016;221(8):4203–19. doi: 10.1007/s00429-015-1161-1 26669874

[94] Ekşi MŞ, Özcan-Ekşi EE. Fatty infiltration of the erector spinae at the upper lumbar spine could be a landmark for low back pain. Pain Pract. 2024;24(2):278–87. doi: 10.1111/papr.13302 37830410

[95] Ekşi MŞ, Öztaş UO, Topaloğlu F, Yeşilyurt SC, Duymaz UC, Osama M, et al. Erector spinae could be the game changer in surgical decision-making in patients with lumbar spondylolisthesis: a cross-sectional analysis of an age-, sex-, subtype-, level-matched patients with similar spinopelvic parameters received surgical or conservative management. Eur Spine J. 2024;33(10):3715–23. doi: 10.1007/s00586-024-08341-3 38809440

